# A scoping review of integrated arts therapies and neuroscience research

**DOI:** 10.3389/fpsyg.2025.1569609

**Published:** 2025-05-08

**Authors:** Rebecca Bokoch, Noah Hass-Cohen, April Espinoza, Tyler O’Reilly, Elad Levi

**Affiliations:** California School of Professional Psychology, Alliant International University, Alhambra, CA, United States

**Keywords:** scoping review, art therapy, arts therapies, neuroscience, modalities

## Abstract

**Introduction:**

This article provided a scoping review of the current state of the field for integrated arts therapies and neuroscience research. The main arts therapies modalities included in this review were: of arts therapies (i.e., drawing, painting, sculpting, bibliotherapy, cinema therapy, dance movement therapies, drama therapies, music therapies, neuroaesthetics, phototherapies, and poetry). The main objectives of this integrated arts therapies and neuroscience scoping review were to: (1) include multiple modalities of arts therapies, (2) summarize, synthesize, compare, and contrast populations, settings, presenting problems, methods, modalities, measures, and outcomes, (3) discuss implications, and (4) suggest future directions.

**Methods:**

The design for this scoping review was conducted according to PRISMA-ScR and the JBI Scoping Review Manual standardized recommendations. Eligibility criteria included: English language, peer-review, integration of arts therapies and neuroscience, and use of research methodologies such as case studies, quantitative, qualitative, mixed methods, systematic or scoping reviews, and meta-analyses. Articles were gathered from two online databases (EBSCOHost and PubMed) using keywords, and review of articles from reference lists. Publications that met criteria were reviewed and charted for the following information: author, year of publication, sample size and characteristics, research design, data analyses, modality (i.e., intervention, treatment), and outcomes. This scoping review included 84 publications that met inclusion criteria, after the research team discussed to consensus.

**Results:**

Outcomes suggested improvements in brain activity and integration, cognitive, affective, sensory, and social functioning, memory reconsolidation, psychological symptoms, affect, and behavior.

**Discussion:**

Interpretations were limited in that most publications lacked directionality in their approach, were exploratory, and dependent on researcher assumptions, expertise, and access to instruments and populations. Therefore, more research is needed on each modality that upholds stronger research methodology, and can develop focus across researchers. While this scoping review was able to summarize and synthesize the state of the field, it is still too early to be able to compare outcomes or make more solid conclusions about specific neuroscientific processes and benefits for each individual modality. This body of knowledge provided valuable implications for the field and made suggestions for future directions.

## Introduction

Integrated arts therapies and neuroscience research has focused on establishing the efficacy of arts-based practices and continues to be an emergent area of interest (Hass-Cohen et al., 2022; Hass-Cohen and Clyde Findlay, 2015; Hass-Cohen and Clyde Findlay, 2019; King and Strang, 2024; Malik, 2022; Strang, 2024; Vaisvaser, 2024). Multiple theoretical chapters and articles have made the connection between arts therapies and neuroscience (Hass-Cohen and Carr, 2008; Hass-Cohen and Clyde Findlay, 2015; Hass-Cohen and Clyde Findlay, 2019; King and Strang, 2024). Reviews of arts therapies and neuroscience have been mostly brief and narrative (Malik, 2022; Oliva et al., 2023). Reviews have also highlighted a need for research that goes beyond theoretical claims and subjective reports (Malik, 2022). Thus, theoretical interpretations have been made without empirical backing, possibly due to a lack of access and understanding of biophysiological instruments in the general arts therapies field. A methodological review process that includes a team approach to analysis to reduce bias and provide a more in depth and valid map of the state of the field, is needed (Malik, 2022). Another constraint of the existing reviews of arts therapies and neuroscience is the research focus on one modality, e.g., art therapy (Malik, 2022) or music therapy (Tramontano et al., 2021), or by one specific population or clinical concern, e.g., Alzheimer’s or dementia (Popa et al., 2021). For almost two decades, there has been a call to further the understanding of neuroscience structures and functions associated with mechanisms of change in arts therapies (Hass-Cohen and Carr, 2008; Hass-Cohen and Clyde Findlay, 2015; Hass-Cohen and Clyde Findlay, 2019; King and Strang, 2024).

For purposes of this scoping review, the field of arts therapies are defined as art therapy and creative and expressive arts therapies (i.e., drawing, painting, sculpting, a variety of media), bibliotherapy, cinema therapy, dance therapy (and other movement therapies), drama therapy (including psychodrama, therapies incorporating performance art), music therapies, neuroaesthetics (including the experience of art, i.e., viewing art), phototherapy (including photography and video), and poetry (American Psychological Association, 2025). From a neuroscience perspective, each modality focuses on one biological system, for example, the visual and sensory system with art therapy, auditory system with music therapy, and motor system with dance therapy.

Arts therapies and neuroscience research has addressed some but not all neuropsychological psychological domains, such as: affect, behavior, cognition, cognitive function (i.e., memory, mindfulness), executive function, interpersonal/relational functioning and developmental neuroscience (i.e., attachment), mental health symptoms and functioning (i.e., anxiety, depression) (Czamanski-Cohen and Weihs, 2016; Malik, 2022; Strang, 2024). According to this research, related neuroscientific structures, neuropathways, and functions, have included: cortical and subcortical nervous system responses (i.e., responses to stress and trauma), endocrine systems, immune systems, movement and motor systems, sensations and perceptions (i.e., visual, tactile, auditory), and other relevant neuroscience information. For this scoping review the advantages of inclusion of multiple modalities for the current scoping review will provide an understanding of the integration of arts therapies and neuroscience and their benefits (Vaisvaser, 2024), as well as present gaps in addressing neuroscientific related functions and neuropsychological domains.

## Methods

This article has provided a scoping review of the current state of the field for integrated arts therapies and neuroscience research. The overarching research question in this study was: “What integrated arts therapies and neuroscience research has been conducted to date?” Research sub-questions were organized according to the following main domains (research and clinical). They were: (1) which research methodologies and instrument were used, and what were the suggestions for future research to strengthen the body of research in this emerging field? (2) What clinical approaches and modalities, as well as populations, settings, and presenting problems have existing arts therapies and neuroscience research studies included, and what were the suggestions for clinical practice? (3) What is the comparative efficacy of different modalities for the various populations, settings, and presenting problems?

Theoretical and conceptual research was initially considered for inclusion in this scoping review; however, they were excluded as they would require an analysis of conceptual frameworks, principles, definitions, and foundations. Such an analysis merits its own scoping review, given the large number of theoretical publications on integrated arts therapies and neuroscience. Subsequently, this scoping review aimed to summarize the outcomes, results, and findings gathered from the more recent and prevalent clinical and empirical publications in this field. Because this review included a variety of research methodologies and aimed to organize the scope of a body of literature as well as identify the gaps in the literature to inform future clinical research, a scoping review was warranted (Munn et al., 2018). This scoping review also aims to create a foundation of knowledge on this integrated field to inform clinical applications, and depository of relevant publications to support further research.

The main objectives of this integrated arts therapies and neuroscience scoping review were to: (1) include main art therapy modalities (i.e., art therapy, dance, drama, movement, music, photo), (2) compare and contrast the efficacy of different art therapy modalities, (3) summarize, synthesize, compare, and contrast the relevant methodologies within the publications (i.e., meta-analyses, systematic or scoping reviews, quantitative, mixed methods, qualitative, case studies), as well as populations, settings, presenting problems, measures, and outcomes, (4) discuss implications, and (5) discuss suggestions for future directions.

### Research design

The design for this scoping review was informed by PRISMA-ScR (Tricco et al., 2018) and the JBI Scoping Review Manual (Peters et al., 2021). Per the definition, the main inclusion criterion was the integration of arts therapies and neuroscience, meaning that clinical arts therapies approaches, as well as neuroscience theories or measures were linked in the publications. Additional inclusion criteria for articles in this scoping review were any case studies, primary research (quantitative, qualitative, mixed methods), systematic or scoping reviews, or meta-analyses articles. There was no limitation on years considered, as it was expected that the state of the field would benefit from a comprehensive review, as most research has been conducted in the past two to three decades. All articles were peer-reviewed publications, provided in English.

### Procedures

Article searches were conducted in two steps. First, two online databases, EBSCOHost and PubMed, were searched using the following keywords and Boolean operators: “arts therapies,” or “expressive arts therapies,” or “art therapy,” and “neuroscience” or “neuropsychology.” There was no limitation on year of publication date, as this scoping review was exploratory, and it was expected that the range of time would not be too broad to be included as integrated arts therapies and neuroscience is a more recent, emergent field. Second, reference lists of articles were searched for additional sources. These article search steps were conducted from April 2024 to October 2024. The research team consisted of clinicians and researchers in the field of arts therapies and neuroscience, and graduate psychology student research assistants with clinical interest in arts therapies. Each article was reviewed via text analysis of titles, abstracts, and keywords by individual researchers, then two subsets of researchers among the team reviewed the article categorizations independently and discussed until consensus was collectively reached by the research team for inclusion.

**Figure 1:**
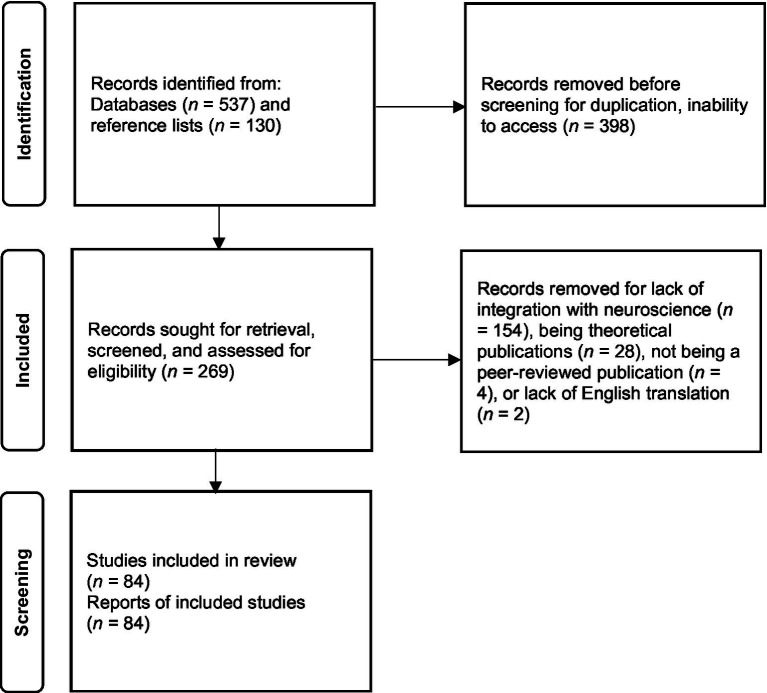
Venn diagram of the six overlapping principles/factors of Art Therapy Relational Neuroscience with partial descriptions. CREATE: (1) creative embodiment in action. (2) relational responding, (3) expressive communicating, (4) adaptive responding, (5) transformative integration, and (6) empathizing and compassion. Partial descriptions from Hass-Cohen and Clyde Findlay (2015).

Initially, 667 articles were found. Then, 269 articles were identified as potentially meeting inclusion criteria. After further team review, 84 articles met inclusion criteria. Exclusion criteria included theoretical articles, and a lack of integration between arts therapies and neuroscience. Research that only peripherally mentioned neuroscience as a theoretical basis or theme, or publications that did not apply neuroscience to the arts therapies, were excluded as they did not provide sufficient information or meet inclusion criteria of integration. Additionally, while neuropsychological conditions and biophysiological instruments are included in these publications, their use alone would not merit inclusion in this scoping review, as eligibility required the integration of arts therapies and neuroscience. Therefore, these inclusion and exclusion criteria prioritized clinical research studies. Publications that met criteria were charted for the following information first by individual researchers and then reviewed by the research team: author, year of publication, sample size and characteristics, research design, data analyses, modality (i.e., intervention, treatment), and outcomes related to the objectives of this scoping review. A discussion focusing on the knowledge generated by this depository, as well as research and clinical impressions concluded this process (Figure 1).

The 84 publications included in this scoping review were organized by research design (Figure 1). There were five review articles, 28 quantitative empirical articles, nine mixed methods articles, four qualitative studies and 38 clinical case study reports (Table 1). Of the included publications study designs: 17 were experimental (20.24%), 11 were quasi-experimental (13.10%), 9 were pre-experimental (10.71%), and 45 were observational (53.57%).

**Table 1:** Article research methodologies.

Article type	Research design	*n*	%
Case study	Quantitative	5	
	Mixed methods	8	
	Qualitative	25	
	Total	38	45.24%
Quantitative	Pretest-posttest experimental control group design	12	
	Pretest-posttest non-equivalent control group design	5	
	Posttest only experimental control group design	1	
	Posttest only non-equivalent control group design	2	
	Pretest-Posttest single group design	5	
	Within subjects pretest-posttest experimental design	2	
	Static comparison group design	1	
	Total	28	34.57%
Mixed methods	Pretest-posttest control group experimental design	2	
	Pretest-posttest non-equivalent control group design	4	
	Pretest-posttest single group design	4	
	Thematic analysis	8	
	Latent content analysis	3	
	Case study description	1	
	Total	9	10.71%
Qualitative	Thematic analysis	3	
	Latent content analysis	1	
	Total	4	4.94%
Review	Scoping review	2	
	Systematic review	2	
	Meta-analysis	1	
	Total	5	5.95%

Most publications were case studies (45.24%). A strength of the case studies was that some of them utilized biophysiological instruments (Belkofer and Konopka, 2008; Fachner et al., 2019; Kang et al., 2022; Pąchalska, 2022; Pąchalska et al., 2013; Pąchalska et al., 2021; Walker et al., 2016), as well as reporting on quantitative (Belkofer and Konopka, 2008; Kang et al., 2022; Kang and Thaut, 2019; Pąchalska et al., 2013) or mixed methods data (Fachner et al., 2019; Guseva, 2018; Pąchalska et al., 2021; Pąchalska and Góral-Półrola, 2020; Pąchalska, 2022; Walker et al., 2016; Warson and Warson, 2023). Other case studies mainly reported on qualitative findings, and shared descriptive case examples and clinical applications. Almost all case studies proposed theoretical conclusions.

Quantitative studies were the second most common type of publication (34.57%), with a range of designs including: pretest-posttest experimental control group design, pretest-posttest nonequivalent control group design, pretest-posttest single group design, and posttest only experimental control group design, posttest only nonequivalent control group design, within subjects pretest-posttest experimental control group design, and static comparison group design. About half of the quantitative studies were experimental, including random assignment to treatment versus control groups (*n* = 15; 53.57%) (Abbing et al., 2019; Bastepe-Gray et al., 2022; Bolwerk et al., 2014; Corbett et al., 2019; De Bartolo et al., 2020; Hass-Cohen et al., 2021; Herrera-Arcos et al., 2017; Iosa et al., 2021; Josef et al., 2019; Kaimal et al., 2020; Kang et al., 2021; Kruk et al., 2014; Pongan et al., 2017; Schindler et al., 2015; Verna et al., 2020), a quarter of the quantitative studies were quasi-experimental (*n* = 7; 25%) (Belkofer et al., 2014; Choi et al., 2009; Costa et al., 2020; Cucca et al., 2021; Hsieh et al., 2019; Kaimal et al., 2017; Walker et al., 2018), and about a fifth of the quantitative studies were pre-experimental (*n* = 6; 21.43%) (Ettinger et al., 2023; Fisher et al., 2020; Goldblatt et al., 2011; Haiblum-Itskovitch et al., 2018; Kleinmintz et al., 2014; Spring, 2004).

Mixed methods studies are the third most common (*n* = 9; 10.71%), with all but one study utilizing thematic analysis (*n* = 8; 88.89%), along with pretest-posttest single group design, pretest-posttest nonequivalent control group design, or pretest-posttest control group design. The least common types of publications were qualitative studies (*n* = 4; 4.94%) using thematic or latent content analysis, as well as review publications (*n* = 5; 5.95%), such as: scoping reviews, systematic reviews, or meta-analyses (Table 1).

## Results

### Review publications

Five review articles were included in this scoping review, including one meta-analysis, two systematic reviews, and two scoping reviews (Table 2). The number of articles included in these review publications ranged from 18 (Griffith and Bingman, 2020) to 46 articles (Malik, 2022; Tramontano et al., 2021). Three review articles focused on adults with neurological disorders or impairments and their caregivers, one focused on varied clinical populations, and one focused on the general adult non-clinical population. Interventions in these review articles included music therapy, drawing, and multiple methods of arts therapies (e.g., art, dance, drama, music). The results gathered from these reviews included neurophysiological and psychological outcomes, as well as implications for future research. Neurophysiological findings showed (1) activation of the prefrontal and cingulate cortices, functional connectivity, and (2) improvements in: cognitive function (e.g., information-processing, visual spatial attention, attention, episodic memory) and motor functions (i.e., walking), as well as cortisol regulation and pain outcomes (Griffith and Bingman, 2020; Malik, 2022; Oliva et al., 2023; Popa et al., 2021; Tramontano et al., 2021). Psychological findings included reduced anxiety, depression, fatigue, and aggression, as well as increased purpose in life, quality of life, self-confidence, socialization, and social status (Malik, 2022; Oliva et al., 2023; Popa et al., 2021; Tramontano et al., 2021). Implications for future research suggested seeking to build upon limitations in the field, such as reducing risk of bias, and conducting more quantitative studies (Table 2).

**Table 2:** Review research.

Author and year	Number of articles	Population	Research design	Interventions	Outcomes
Griffith and Bingman (2020)	18 articles	General adult	Meta-analysis	Art-based interventions: drawings (internally and externally cued tasks)	Internally cued drawing was associated with prefrontal and cingulate cortices activation. Externally cued drawing was not found to be associated with activation of ventral visual pathways and the temporal lobe.
Malik (2022)	46 articles	Varied	Systematic narrative review	Art therapy	This review found positive outcomes for trauma, psychological, neurological, and injury related issues using art media such as clay, paint, drawing, sculpture, mask-making, sand tray, and collage. Neurological outcomes were collected using EEG, fNIRS, and fMRI, and discussed changes in brain structures, functions, and pathways related to visual, motor, emotional, somatosensory, and memory.
Oliva et al. (2023)	26 articles	Neurorehabilitation with adult clinical populations (Parkinson’s, stroke, acquired brain injury)	Systematic mini-review	Multiple arts therapies: art, dance, drama, and music	They found that only 5 of the studies had a low risk of bias and that art was the most commonly used modality. The articles have significant results in functional connectivity, reducing anxiety/depression, purpose in life, self-confidence, and reducing fatigue in therapy but not significant in terms of ADLs. They also noted that there were twice as many qualitative/feasibility studies than quantitative.
Popa et al. (2021)	20 articles	Alzheimer’s dementia patients and their caregivers	Scoping review	Multiple arts therapies: music therapy (calming music, music-based physical exercise, etc.) and art therapy (painting, drawing, etc.)	The review found benefits of both music therapy and art therapy on quality of life of patients with Alzheimer’s dementia, with the best effects related to increased socialization and the maintenance of social status. The findings showed that art therapy improved domains of cognitive functioning for patients with AD, including information-processing, visual spatial attention, attention, and episodic memory. Music therapy was found to reduce cortisol levels of patients with AD, which as a result reduced symptoms of anxiety, depression, and aggression.
Tramontano et al. (2021)	46 articles	Individuals with neurological disorders (multiple sclerosis (MS), stroke, Parkinson’s disease (PD), traumatic brain injury (TBI), Cerebral Palsy (CP), etc.)	Scoping review	Music-based interventions: Music-based therapy (Melodic Intonation Therapy (MIT); Musical Sensory Orientation Training (MSOT); Musical Attention Control Training (MACT), etc.)	This review highlighted the potentiality and the versatility of the music-based therapy in the rehabilitation of neurological disorders. Cognitive and motor abilities, mood disorders, quality of life, the reduction of pain, and perceived fatigue seem to be modifiable by different music therapy techniques. Several studies have shown that the use of music therapy improves walking in patients with MS, stroke, PD and CP.

### Quantitative research review

The 28 quantitative articles were reviewed for methodological and statistical strengths and weakness associated with random assignment to groups, inclusion of control or comparison groups, sample size, instruments, and statistical analyses. Arts therapies interventions were also reviewed for modality, frequency, and duration. A summary of results and recommendations followed (Table 3).

**Table 3:** Empirical articles summary table.

Author and year	Sample size	Population	Research design	Data analysis	Interventions	Outcomes
Abbing et al. (2019)	47 (47F, mean age = 44.4)	Adults; Primary diagnosis of GAD, social phobia or panic disorder	Pretest-posttest experimental control group design	RANOVA	Art-based intervention: Anthroposophic art therapy (10- to 12-h long sessions; waitlist control group)	Anxiety symptoms were significantly reduced in the experimental group (AT). The AT group demonstrated higher HRV after treatment (lower stress level and/or reduction of anxiety). However, stress response remained unchanged after intervention. Significant improvements in some areas of executive functioning (EF): emotion control, working memory, plan/organize and task monitor. Areas of EF that were not significant in comparison to the control were inhibit, shift, self-monitor, initiate and organization of material. Emotion control, plan/organize and task monitor were also associated with stress reduction while the others were not.
Bastepe-Gray et al. (2022)	26 (17 M, 9F; mean age = 67.22)	Parkinson’s patients	Pretest-Posttest experimental control group design	RANOVA	Music-based intervention: Guitar instruction, 6 weeks of twice a week 1-h instruction implemented by music teachers at a community music school. Focused on finger isolation, reach and grab velocity, and eye-hand coordination timing and accuracy.	Significant improvement in BDI-II, PDQ-39, mood, depression, quality of life, and anxiety for the early intervention group but no improvements in QoL, mood, or anxiety for the late intervention group.
Belkofer et al. (2014)	10 (3 M, 7F; ages 24–49)	Young adults; artists and non-artists	Pretest-posttest non-equivalent control group design	Dependent samples *t*-test	Art-based intervention: Drawing with oil pastels, 20-min single session. Guided by research assistants.	For artists, significant alpha activity was found in the posterior visual/spatial areas of the cortex. For non-artists, differences in alpha activity in the right parietal and right prefrontal areas. There were no significant findings between the groups. Beneficial implications for art therapy, as alpha rhythm is associated with self-regulation, relaxation, memory, visual processing.
Bolwerk et al. (2014)	28 (13 M, 15F; mean age = 63.71)	Post-retirement adults	Pretest-posttest test experimental control group design	Wilcoxon signed-rank tests; Analysis of covariance (ANCOVA)	Art-based intervention: drawing, painting, etc. Neuroaesthetics: cognitive evaluation of art (selected paintings and sculptures). 2-h sessions, 1x/week, 10 weeks.	Art production group showed greater spatial improvement in functional connectivity of posterior cingulate cortex/precuneus to frontal and parietal cortices from pretest to posttest than the cognitive evaluation of art group. Functional connectivity in the art production group was related to psychological resilience post-intervention.
Choi et al. (2009)	20 (4 M, 16F; mean age 77.4)	Dementia	Posttest only non-equivalent control group design	Independent samples *t*-test	Music-based intervention: 50 min, 2x/week, 5 weeks.	Music intervention group showed significant improvement with regard severity of symptoms, especially agitation. There were also beneficial effects of music intervention on caregiver distress and agitation. There were no significant differences following treatment between the two groups.
Corbett et al. (2019)	77 (18F, 59 M; EXP mean age = 11.12; WLC mean age = 11.83)	Children with ASD	Pretest-posttest experimental control group design	Independent sample *t*-tests, ANCOVA	Performance arts-based intervention	Experimental group demonstrated better verbal theory of mind skills, social motivation, and ability in repeated face task. TOM contextual subtest was not significant.
Costa et al. (2020)	16 (12 M, 4F; mean age = 45.9)	Schizophrenia	Pretest-posttest non-equivalent control group design	Mann-Whitney *U* test	Art-based intervention: Two weekly 90-min sessions for 3 months of art activities (blind drawing, reversed drawing, void design).	No evidence that guided methods boost cognitive effects. Psychosocial/affective benefits were enhanced by unguided methods, suggesting that therapeutic methods can make a difference. No cross-group differences for cognitive effect.
Cucca et al. (2021)	27(8 M, 19F; mean age = 67.4)	Parkinson’s	Pretest-Posttest nonequivalent control group design	Independent samples *t*-test; Dependent samples *t*-test	Art-based intervention: Clay manipulation, painting on canvas, drawing, etc. 20 sessions, 90 min each, 2x/week, 10 weeks. Guided by credentialed art therapist.	Analyses of fMRI showed increased functional connectivity within dorsal attention (DAN) and executive control (ECN) brain networks in patients compared to controls. Following art therapy, performance improved on Navon test, eye tracking, and UPDRS scores. fMRI analysis revealed significantly increased functional connectivity levels in brain regions within V1 and V2 networks.
De Bartolo et al. (2020)	60 (30 M, 30F; Parkinson’s group mean age = 72.5, elderly adult group mean age = 72.1, young adult group mean age = 32.3)	Parkinson’s	Pretest-posttest experimental control group design	Mixed ANOVA	Music and movement-based intervention: Listening to six different music tracks while walking an 18-m corridor. 14 trials total. Recorder for each participant (first trial without music, six trials with six tracks, then 7 trials reversed ordered). Instruction provided by researchers.	The main effect of music tracks resulted statistically significant in all the gait parameters (*p* < 0.05), but for symmetry of lower trunk movements. This effect was independent by group. The only significant interaction between music and group, in fact, was found for pelvis obliquity range of motion (*p* = 0.019) *Post-hoc* analyses showed as classical music reduced speed and trunk tilting (*p* < 0.01), whereas the range of pelvic obliquity movements in frontal plane were increased by rock, motivational, and heavy metal songs (*p* < 0.015).
Ettinger et al. (2023)	42 (12 M, 30F; mean age = 68.4)	Parkinson’s	Pretest-posttest single group design	Dependent samples *t*-tests; Wilcoxon signed rank tests	Art-based intervention: H-T–P assessment, 9 art projects changing bi-weekly including painting, drawing, sculpture, and group projects. 20 sessions, 2x/week, 90-min sessions, 10 weeks.	Significant improvements in all aspects of HTP-PDS which includes motor control, visual/spatial function, cognition, motivation, emotion, self, interpersonal, creativity, and global functioning.
Fisher et al. (2020)	11 (5 M, 6F; mean age = 65.8)	Mid-Stage to severe Parkinson’s	Pretest-posttest single group design	Wilcoxon signed rank test	Dance and movement-based intervention: 10 weeks of 1.5-h dance movement therapy with emphasis on improvisation led by dance movement therapists.	Significant increase in balance and cognition. 13% increase in overall motor function, no significant changes in social cognition or gesture production.
Goldblatt et al. (2011)	22 (16 M, 6F; mean age = 71.4)	Parkinson’s	Pretest-posttest single group design	RANOVA	Art-based intervention: Single session intervention of clay sculpting, 40 min.	Significant decrease in obsessive compulsive measure, depression measure, and phobic anxiety measure.
Haiblum-Itskovitch et al. (2018)	50 (26 M, 24F; mean age = 33)	Adults	Pretest-posttest single group design	RANOVA	Art-based intervention: Three, 10 min art making sessions (pencil, oil-pastels, and gouache paint), 10 min.	Drawing with gouache paint and oil-pastels resulted in increased positive mood (self-report measure). Largest suppression of PNS and augmentation of SNS occurred during art making with oil pastels. No linear other relationship was found between emotional state and HRV parameters.
Hass-Cohen et al. (2021)	34 (6 M, 28F; mean age = 35)	Adults with chronic pain	Pretest-posttest experimental control group	Mixed RANOVA	Art-based intervention: Three- and four- drawing protocols, single session, 2 h. Guided by researchers.	Significant improvements in ratings of pain, depression, anxiety, relationship quality, and helplessness from pretest to posttest. Endorsement of all resource areas significantly improved from pretest to posttest and pretest to follow up. There were significant differences between the three- and four-drawing protocol groups for ratings and frequency of anger and participants in the resource reminder condition had lower anger ratings than participants in the no resource reminder condition. There was a greater reduction in frequency of experiencing fear over time in the no resource reminder condition than in the resource reminder condition. There were no other significant between group differences or interaction effects.
Herrera-Arcos et al. (2017)	209 (86 M, 123F; age range = 6–88)	Children and adults	Posttest only experimental control group design	Independent samples *t*-test	Neuroaesthetics: Viewing artistic stimuli in an art exhibit (paintings), 1–2 min per art piece. Guided by museum guides.	Suppression of beta band frequencies in the prefrontal electrodes during appreciation of subject’s favorite painting, as measured by EEG. No significant differences in brain activity in relation to the presence or absence of explanation during exhibit tours were found.
Hsieh et al. (2019)	24 (5 M, 19F; mean age = 68.3)	Adults	Pretest-Posttest non-equivalent control group design	Dependent samples *t*-test; Independent samples *t*-test	Movement and mindfulness-based intervention: Movement therapy (exercise), hand-eye coordination training (Chinese calligraphy), meditation. 3 h (1 h each modality), 2x a week, 16 weeks.	Significant improvements in senior fitness tests, significant decrease in depression scores, no significant results found based on demographics, Subjective Memory Complaint (SMC) group had lower cognitive function and higher depressive scores compared to non-SMC group.
Iosa et al. (2021)	24 (13 M, 11F; EXP1 (*n* = 20) mean age = 30.2; EXP2 (*n* = 4) mean age = 59.5)	EXP1: 20 healthy adults; EXP2: 4 adults with stroke	Within subjects pretest-posttest experimental design	EXP1 RANOVA; Wilcoxon signed ranks test; EXP2 Dependent samples *t*-test	Neuroaesthetics: Viewing paintings in virtual reality.	EXP1: Healthy subjects exposed to art masterpieces completed the task with shorter hand pathways and lower perception of physical demands; EXP2: Patients exposed to the art masterpieces showed significant in all computed parameters, especially in reduction of errors.
Josef et al. (2019)	62 (62 M; mean age = 26.3)	Adults	Pretest-posttest experimental control group design	Independent sample *t*-test; simple linear regression.	Dance-based intervention: Dancing with oxytocin for treatment group.	Oxytocin improved movement synchrony in dancing pairs and this increase was of a greater degree for those with higher empathic abilities.
Kaimal et al. (2017)	26 (11 M, 15F; mean age 32.5)	Adults	Pretest-posttest non-equivalent control group design	Repeated measures analysis of variance (RANOVA)	Art-based intervention: Three drawing conditions (coloring mandala, doodling, free drawing) presented for 5 min (2 min eyes closed).	Coloring, doodling and free drawing all activated the medial prefrontal cortex and reward pathway. Positive outcomes in self-perception of creativity.
Kaimal et al. (2022)	24 (6 M, 18F; mean age = 27.5)	Adults (mean age = 27.5)	Within subjects pretest-posttest experimental design	Mixed repeated measures analysis of covariance (RANCOVA)	Art- and sensory-based intervention: Drawing in virtual reality, aromatherapy.	Significant differences in PFC activation between rote tracing task and artmaking task.
Kang et al. (2021)	29 (18 M, 11F; EXP mean age - 10.5; CON mean age = 9.2)	Children (7–13 years)	Pretest-posttest experimental control group design	Independent samples *t*-test; Dependent samples *t*-test	Mindfulness and art-based intervention: Nature-based arts, sculpting, phototherapy, collage, mindfulness, and drawing.	Activities in the forest increased alpha and theta waves. High ATQ was observed as well, indicating alertness and immune function. Participants also demonstrated increased stress reduction and self-esteem levels at posttest.
Kleinmintz et al. (2014)	131 (68 M, 63F; mean age = 24.7)	Adults	Static comparison group design	MANOVA	Music-based intervention	Musicians trained in improvisation are more creative than those without improvisation training and non-musicians. Evaluation mediates the relationship between time invested in improvisation, fluency, and originality.
Kruk et al. (2014)	14 (14F; mean age = 23.8)	Medical school students	Pretest-posttest test experimental control group design	Mixed RANOVA	Art-based intervention: Clay sculpting and drawing. Single session, 30 min. Guided by researchers.	Both interventions increased gamma power in the right medial parietal lobe compared to general movement. Clay sculpting decreased right medial frontal gamma power and elevated theta power. There were no statistically significant differences between the two frontal electrodes, F3 versus F4, or between the two parietal electrodes, P3 versus P4.
Pongan et al. (2017)	59 (20 M, 39F; singing group (*n* = 31) mean age = 78.8; painting group (*n* = 28) mean age = 80.2)	Probable Alzheimer’s disease according to DSM-V criteria	Pretest-posttest experimental control group design	Independent samples *t*-test; Mann- Whitney U; Chi-squared goodness of fit; Mixed RANOVAs	Music- and art-based intervention: 12 weekly 2-h singing or painting sessions over 3 months.	Significant decrease in pain for both interventions, reduction in depressive symptoms in painting group, greater improvement in verbal memory in singing intervention, pain scales did not differ between groups, working memory improved in both groups, change in self-esteem did not reach statistical significance.
Schindler et al. (2015)	113 (38 M, 75F; mean age 52.3)	Adults with subjective memory complaints (SMC)	Pretest-Posttest experimental control group design	RANOVA	Art-based intervention: drawing. Neuroaesthetics. 1x/week for 10 weeks. Guided by artist.	Significant time effects were found for processing speed and visuo-spatial cognition. Significant improvement in processing speed and visuo-spatial cognition. SMC participants showed no improvement of processing speed but remained on a relatively low level regarding their performance. No time x art intervention effect was found.
Spring (2004)	45 (45F; age range = 21–45)	Sexual assault victims with diagnosed PTSD	Pretest-posttest single-group design	RANOVA	Art-based intervention: Drawing series (5 drawings, 2 self-portrait concepts, and 3 about relation to the world). Single session. Each drawing was followed by an audiotaped discussion.	Analyses looked at occurrence of shapes in drawings (wedges, eyes, and combinations) between rape only group, multiple sexual abuse, and control groups. Differences in shape occurrence were connected to guilt and feelings of threat.
Verna et al. (2020)	30 (13 M, 17F; mean age = 57.5)	Stroke patients	Pretest-posttest experimental control group design	Wilcoxon signed ranks test; Mann-Whitney *U* test	Music-based intervention: Temporal, negative, mismatched-based music therapy. 3x/week, 4 weeks, 20 min.	Improved quality of life and independence, and lower disability ratings for both control and experimental group. Improved quality of life and independence; reduction in disability ratings for participants in the mismatched based therapy group.
Walker et al. (2018)	10 (10 M; mean age = 42.6)	Chronic mTBI	Posttest only non-equivalent control group design	Mann- Whitney U tests	Art-based intervention: Sculpting, drawing, painting, and mask making.	Participants who expressed patriotic themes in masks reported less PTSD and related symptoms. Injured themed group demonstrated increased neuronal connectivity in the thalamic region of the brain.

#### Research design

A strength was that a majority of the empirical studies included a control group or comparison group (*n* = 22; 78.57%), and almost half of the publications included random assignment to treatment vs. control groups (*n* = 15; 53.57%).

#### Sample characteristics

Sample sizes ranged from 10 to 209 participants, which is very broad. Participant samples included an almost equal number of females and males, more white participants than participants of other ethnicities, and diverse ages ranging from 6 to 88 years old. Presenting problems included neurological disorders (i.e., Alzheimer’s, Parkinson’s, dementia, stroke) (*n* = 12; 42.86%), neurodevelopmental disorders (i.e., autism, developmental disabilities) (*n* = 1; 3.57%), chronic illness (i.e., injury, chronic pain, cancer) (*n* = 2; 7.14%), psychological functioning (i.e., depression, anxiety, schizophrenia, emotion regulation) (*n* = 2; 7.14%), trauma, grief, and resilience (*n* = 1; 3.57%), parent–child relationships, substance abuse, and other general adult and community samples (*n* = 10; 35.71%). About half of the quantitative publications were neurological disorders, and about a third were general adult community samples. There were slightly more female participants than male participants across these studies (56% female), and more white participants than participants of other races and ethnicities, which is a typical limitation in most social science research.

#### Measures and instruments

A diversity of instruments, measures, and assessments were reported, including: (1) biophysiological instruments, (2) neuropsychological and psychological standardized scores from cognitive, perceptual, and performance-based tasks, and (3) standardized psychological assessments.

Biophysiological instruments included: electroencephalogram (EEG), FFT-EEG, qEEG (Belkofer et al., 2014; Corbett et al., 2019; Herrera-Arcos et al., 2017; Kang et al., 2021; Kruk et al., 2014), which measured neuronetwork activation in response to artmaking. Other examples of documenting the response to artmaking included: Event Related Potential (ERP) and Attention Quotient (ATQ). Other approaches included: functional magnetic resonance imaging (fMRI) (Bolwerk et al., 2014; Walker et al., 2018), functional near-infrared spectroscopy (fNIRS) (Kaimal et al., 2017; Kaimal et al., 2020), as well as bioelectrical measures of stress responses, heart rate, and heart rate variability (Abbing et al., 2019; Haiblum-Itskovitch et al., 2018).

There were a wide variety of neuropsychological and psychological standardized measures which included cognitive, perceptual, and performance-based tasks. Examples in alphabetical order included: Amsterdam Neuropsychological Tasks (ANT) (Abbing et al., 2019), Movement Disorder Society sponsored revision of the Unified Parkinson’s Disease Rating Scale (MDS-UPDRS), Navon Test (Cucca et al., 2021), NEPSY subtests (Corbett et al., 2019), Neuropsychiatric Inventory-Questionnaire (NPI-Q) (Choi et al., 2009), Montreal Cognitive Assessment (MoCA) (Costa et al., 2020; Cucca et al., 2021), Stroop test (Costa et al., 2020), Cognitive Assessment Screening Instrument (CASI) (Hsieh et al., 2019), etc. Physiological tracking of gaze (Cucca et al., 2021), gait (De Bartolo et al., 2020), balance (Fisher et al., 2020), and motion (Josef et al., 2019) was also used.

Examples of common standardized psychological assessments used included: Adverse Childhood Experiences Scale (ACES) (Hass-Cohen et al., 2022), Beck Depression Inventory-II (BDI-II) (Bastepe-Gray et al., 2022), Center for Epidemiologic Studies Depression Scale (CES-D) (Hsieh et al., 2019), Hospital Anxiety and Depression Scale (HADS) (Costa et al., 2020), State–Trait Anxiety Inventory (Kruk et al., 2014; Pongan et al., 2017), etc. (Table 3).

#### Statistical analyses

For small sample sizes, some analyses used non-parametric statistical tests to protect against violation of assumptions and low statistical power; thus, the statistical conclusion validity of some of the studies is questionable.

#### Outcomes

Results from biophysiological instruments and measures showed significant changes. Structurally, there were changes in activation in visual/spatial areas of the cortex, specifically for art therapy. Other structural and functional changes in activations in response to all arts therapies reviewed included: the right parietal and prefrontal cortex, gamma power in the right medial parietal lobe, alpha and theta waves, and suppression of beta band frequencies, suggesting reduced demand on cognitive functioning and increased parasympathetic functioning (i.e., relaxation) (Belkofer et al., 2014; Bolwerk et al., 2014; Corbett et al., 2019; Cucca et al., 2021; Fisher et al., 2020; Haiblum-Itskovitch et al., 2018; Herrera-Arcos et al., 2017; Hsieh et al., 2019; Kaimal et al., 2020; Kang et al., 2021; Kruk et al., 2014; Schindler et al., 2015). Spatial improvements in functional connectivity of the default mode network associated with creativity (i.e., posterior cingulate cortex/precuneus to frontal and parietal cortices) were noted for art therapy interventions, specifically, drawing (Bolwerk et al., 2014; Ettinger et al., 2023; Kaimal et al., 2020; Schindler et al., 2015). Executive functioning was found to improve in response to arts therapies (i.e., drawing, clay) based on functional connectivity within dorsal attention and executive control networks (Abbing et al., 2019; Cucca et al., 2021; Kaimal et al., 2017; Kaimal et al., 2020). Sensory function, as related to increased neuronal connectivity in the thalamic region was found (Walker et al., 2018). Motor movements and control, as well as balance, were also improved (De Bartolo et al., 2020; Ettinger et al., 2023; Fisher et al., 2020). In response to art therapy, nervous system activation associated with parasympathetic systems showed positive changes, such as through heart rate variability (HRV) measures (Haiblum-Itskovitch et al., 2018). A summary of quantitative biophysiological measures and instrument outcomes included improvements in theory of mind activation, cognition, motivation, global functioning, alertness and immune function, processing speed, and relaxation.

Results from standardized psychological measures suggested that arts therapies significantly improved anxiety symptoms, executive functioning, depression, quality of life, obsessive compulsive symptoms, helplessness, relationship quality, stress, self-esteem, pain, memory, dementia symptoms, independence, disability ratings, positive affect, creativity, and PTSD symptoms (Abbing et al., 2019; Bastepe-Gray et al., 2022; Choi et al., 2009; Ettinger et al., 2023; Goldblatt et al., 2011; Haiblum-Itskovitch et al., 2018; Hass-Cohen et al., 2021; Hsieh et al., 2019; Kaimal et al., 2017; Kang et al., 2021; Kleinmintz et al., 2014; Pongan et al., 2017; Spring, 2004; Verna et al., 2020; Walker et al., 2018).

#### Modalities

Modalities included arts psychotherapy (i.e., drawing, painting, clay, collage, sculpture, mask making, phototherapy), music-based interventions, dance movement therapy, mindfulness, performance arts, and neuroaesthetics. The frequencies ranged from single to 12 sessions, and the duration varied from 1-min exposures to interventions to 12-h long sessions that took place 1x to 2x per week (Table 3).

### Mixed methods research

There were nine mixed methods studies reviewed (Table 4). Participants included Parkinson’s disease patients (*n* = 4; 44.44%), cancer patients (*n* = 2; 22.22%), adults impacted by trauma (*n* = 2; 22.22%), and community adult subjects (*n* = 2; 22.22%). Across studies there was a similar balance of males and females. Samples ranged in size from 7 to 50, which was small for the tests that were used. Due to small sample size and limited advanced statistical analyses (most used multiple dependent samples-tests or Wilcoxon signed ranks tests; thus, increasing likelihood of error), the validity and generalizability of these results should be interpreted with caution. Almost all studies utilized art-based interventions specifically drawing and clay work. One described a drama-based intervention. The amount of time dedicated to the intervention varied from single sessions lasting for about 45 min each, to weekly sessions lasting from 6 to 12 weeks for 1 to 2 h per session.

**Table 4:** Mixed methods research.

Author and year	Sample size	Population	Research design	Data analysis	Interventions	Outcomes
Bar et al. (2021)	50 (50–87 years)	Parkinson’s disease	Posttest only quasi-experimental non-equivalent control group design; thematic analysis	MANCOVA; thematic analysis	Dance-based intervention: 1x/week for 3 months, contemporary dance classes compared to verbal support control group	Significant differences in general and emotional quality of life, as well as psychological flexibility, where dance group was higher than control group. No significant differences in creative self-efficacy. Open-ended survey questions suggested qualitative support for perceived felt quality of life by the participants.
Czamanski-Cohen et al. (2019)	15 (15F, age range: 36–45 (*n* = 1), 46–70 (*n* = 12), 70 + (*n* = 2))	Breast cancer	Pretest-posttest experimental control group design; thematic analysis	Dependent samples *t*-tests; thematic analysis	Art-based intervention: Eight weekly group sessions. The experimental group (90 min) underwent 10 min of rapport building, 50 min of drawing with a variety of materials during these sessions and emotional processing/discussion, which was based on the Bodymind model of art therapy. The control group (60 min) differed as they spent 45 min coloring mandalas, followed by a 15-min self-care lecture.	Both groups reported benefits from the study but only the experimental group reported benefits on processing difficult emotions related to breast cancer. There were large differences in effect size between the two groups and over time in the pre and post measures of emotion, emotional awareness, and depressive symptoms.
Elkis-Abuhoff et al. (2008)	41 (28 M, 13F, mean age = 70.6)	Parkinson’s disease	Pretest-posttest quasi-experimental non-equivalent control group design; thematic analysis	Dependent samples *t*-test; thematic analysis	Art-based intervention: Single session of clay manipulation guided by researchers. Participants had unlimited time and were directed to squeeze the clay ball 10 times in each hand, and to put the pieces of clay together into a shape other than a ball.	Significant decrease in somatic and emotional symptoms, as measured by the Brief Symptom Inventory (BSI) in both participant groups (the 2 groups were participants with Parkinson’s disease and without Parkinson’s disease). Qualitative analysis of the interviews showed that participants reported a positive emotional response to the clay manipulation, which was reflected in their artwork.
Elkis-Abuhoff et al. (2013)	7 M (mean age 68.2)	Parkinson’s disease	Pretest-posttest single group design; qualitative case example	RANOVA	Art-based intervention: 6 weeks of group art therapy with clay manipulation.	Decrease in all levels of depression, obsessive compulsive thinking, phobia and stress but did not meet statistical significance. Case example demonstrated the impacts of group art therapy where participants were able to explore and express emotions.
Elkis-Abuhoff and Gaydos (2018)	Phase 1 & 2: 41 (EXP: 22, 16 M, 6F, mean age = 71.4; CON: 19, 12 M, 7F, mean age 69.7); Phase 3: 8 (7 M, 1F, mean age = 68.2)	Parkinson’s disease	Phase 1 and 2: Pretest-posttest nonequivalent control group experimental design; thematic and content analysis; Phase 3: Pretest-posttest single group design	Phase 1: Dependent samples *t*-tests; thematic and content analysis; Phase 2: One sample *t*-tests; thematic and content analysis; Phase 3: RANOVA	Art-based intervention: Phase 1- Participants instructed to squeeze clay 10 times with each hand, pull apart and create forms other than balls. Phase 2- Quantitative exploration of data from Phase 1. Further explored in another study. Phase 3- Six weekly, 1-h sessions using a variety of clay types with different topics each week.	Phase 1: Both groups benefited from the Clay manipulation. However, the PD group experienced more benefit in reduction of symptomatology. Phase 2: Found further support for decrease identified in Phase 1 and showed comparable levels to normative adult levels of the BSI. Phase 3: Consistent decrease in all areas of the modified BSI scale but not significant from pre-post weeks 1, 3 and 6.
Elkis-Abuhoff et al. (2022)	Phase 1: 18 (10 M, 8F) Phase 2: 41(5 M, 36F)	Adults	Pretest-posttest quasi-experimental non-equivalent control group design; thematic and content analysis	RANOVA; thematic and content analysis	Art-based intervention: Phase 1- Separated into two groups, drawing and collage, in 45-min session. Phase 2- open time (45 min) to create with all available materials asked to recreate a place in nature they enjoy, then asked to write about their experience.	The greatest increase in positive affect came from participants who lived in urban areas, satisfaction with life scale showed larger increases in satisfaction post-intervention. Qualitatively recurring themes of water, pathways, mountains, and forests were found. Themes in word choices were found where common positive words included interested, inspired, strong, attentive. Negative words were found such as irritable, nervous, and jittery.
Hass-Cohen et al. (2018)	31 (27F, 4 M, mean age = 29.68)	Adults, Trauma	Pretest-posttest single group design; thematic and content analysis	Wilcoxon’s signed rank tests; thematic and content analysis	Art-based intervention: All participants completed four drawings using assorted materials and wrote a title and story for each drawing. The directives consisted of the problem, a self-portrait, resources that helped the problem, and another self-portrait. Single session, 60- to 90-min.	Significant decreases in ratings of trauma and negative affect were found. The decreases in negative affect were identified at posttest and maintained at the two-week follow up. The protocol and inquiry had a self-reported positive impact on participants’ understanding and meaning-making of the traumatic event.
Kaimal et al. (2020)	22 (gender was not reported, mean age = 61)	Cancer patients	Pretest-posttest experimental control group design; thematic analysis	Mixed RANOVA and Wilcoxon signed rank tests; thematic analysis of survey data, use of *a priori* coding scheme	Art-based intervention: Single-session, 45 min. The sessions consisted of either independent coloring (control condition) or open studio art therapy. Facilitated by trained art therapists.	Open art studio and independent color were equally effective in increasing positive affect, self-efficacy, and creative agency, and decreasing negative affect, perceived stress, and anxiety. No significant differences based on condition over time. Participants’ narrative responses supported quantitative themes (feeling more positive, feeling more relaxed, sense of agency), and highlighted additional positive themes such as meaning-making, desire for artmaking in future, addressing aspects of the illness experience, as well as condition-specific themes such as reminiscing, existential reflection, and straightforward distraction.
Munjuluri et al. (2020)	13 (8 M, 5F) mean age (48.2)	Adults impacted by Hurricane Harvey, PTSD	Pretest-posttest single group design; thematic analysis	Dependent samples *t*-test; Thematic analysis	Performance-based intervention: 6 weekly 2-h sessions. Weeks 1 and 6 included fMRIs, weeks 2–5 included Playback theatre performances with informal discussions following each performance.	Initial self-reports showed mild anxiety, PTSD, and depression. Posttests demonstrated decreases in all 3 measures with depression being the only non-significant change. Qualitative findings showed event themes of denial, preparation, clean-up, rescues, rebuilding, and coming back. These were paired with emotional themes of shame, guilt, resentment, and puzzlement.

Results from the quantitative data in mixed methods studies included improvements in life satisfaction, positive affect (Elkis-Abuhoff et al., 2022; Kaimal et al., 2020), quality of life, psychological flexibility (Bar et al., 2021), processing difficult emotions (i.e., depression) and emotional awareness related to cancer (Czamanski-Cohen et al., 2019), decrease in somatic and emotional symptoms (Elkis-Abuhoff et al., 2008; Elkis-Abuhoff and Gaydos, 2018), such as OCD, phobias (Elkis-Abuhoff et al., 2013), and stress (Elkis-Abuhoff et al., 2013; Kaimal et al., 2020), trauma (Hass-Cohen et al., 2018; Kaimal et al., 2020), depression, and negative affect (Elkis-Abuhoff et al., 2013; Hass-Cohen et al., 2018; Kaimal et al., 2020; Munjuluri et al., 2020). Findings from qualitative data suggested positive emotional response to the art interventions (Elkis-Abuhoff et al., 2008), emotional expression of both negative and positive emotions (Elkis-Abuhoff et al., 2013; Elkis-Abuhoff et al., 2022; Kaimal et al., 2020; Munjuluri et al., 2020), meaning making (Hass-Cohen et al., 2018; Kaimal et al., 2020), perceived improvements to felt quality of life (Bar et al., 2021), and addressing aspects of the illness experience (Kaimal et al., 2020) (Table 4).

### Qualitative research

There were four qualitative studies included in this scoping review. The methodologies of the qualitative studies included thematic analysis (*n* = 3; 75%) and latent content analysis of underlying meaning (*n* = 1; 25%). Data included transcripts of a group therapy session, individual interview transcripts, qualitative survey responses, drawings/artwork, and video recorded observations. Participants included: experienced art therapy clinicians, art therapy graduate students, adults with chronic pain, veterans with PTSD, and the general adult population. Sample sizes ranged from 5 to 122 participants that were majority female (80–100%), although the study with five veterans only included male participants (100%). All interventions involved single session art therapy individual or group experiences that incorporated drawings and mandalas after hypnotic induction, attachment bookmaking using cloth, three- and four-drawing protocols, and use of clay and pastels. Not all publications reported the length of the intervention, but of those reported, sessions lasted 2 h.

The main findings from these qualitative studies included emergent themes of (a) the neuroscientific aspects of attachment-based respect, care, and support (Hass-Cohen et al., 2015), (b) neurobiological processes associated with PTSD treatment specifically disconnection vs. connection, opening up as revealed by autobiographical retelling (Lobban, 2014), (c) Examination of neuroscience-based protocols for the amelioration of physical pain and stress which also reported on changes in cognitive/affective, social functioning, and self-identity (Hass-Cohen et al., 2022), and (d) the creation of an art therapy neuroscientific safety assessment tool based on the interpretation of pictural latent content (Gerge, 2017). In summary, the qualitative findings suggested the utility of arts therapies interventions for the amelioration of neuropsychological stress, threat and pain related syndromes and strengthened psychosocial capacities (Table 5).

**Table 5:** Qualitative studies.

Author and year	Sample Size	Population	Research design and analysis	Interventions	Outcomes
Gerge (2017)	122 (80% F)	Experienced clinicians	Latent content analysis of drawings	Art-based intervention: Analyzed 244 pictures drawn by clinicians about worrying/reassuring sessions with a client; Subgroup created mandala drawings before and after a hypnotic induction.	Pictorial signs identified to look for neuroception of safety, neuroception of ambivalence/worry/fight-flight responses, and neuroception of life-threats/submission. Assessment tool created based on findings.
Hass-Cohen et al. (2015)	22 (100% F)	General; graduate art therapy students	Thematic analysis of a post-workshop survey, video/observational analysis of non-verbal touch and communication during workshop	Art-based intervention: Single session group workshop in which participants utilized personal fabrics scraps and felt-fabric sheets to create an Attachment-Based Cloth album. After creating the albums, the group divided into pairs and shared their album, and each member reported on their partner’s album back in the large group.	Survey analysis revealed the following themes: respect, care, and support, the degree of which was mediated by familiarity. These themes were facilitated by art mediated intra- and interpersonal touch and space.
Hass-Cohen et al. (2022)	25 (82.4% F)	Adults between ages 19 to 66 with chronic pain	Thematic analysis of interviews as well as art titles and characteristics	Art-based intervention: Three- or four-drawing protocol, single session, lasting 2 h.	Themes included changes across interviews as well as drawing titles and characteristics in the following areas: cognitive/affective functioning, perceived social support, sense of control over pain, stress levels, psychological processing, and self-representation/identity.
Lobban (2014)	5 (100% M)	Veterans with PTSD	Thematic analysis of the veterans’ autobiographical accounts and artwork	Art-based intervention: Single session groups, 2 h, led by an art therapist. The first hour consisted of image making (including molding clay, pastels, drawing), and the second hour consisted of discussion and reflection with the entire group.	Several themes emerged from the group, including: identified problem areas around disconnection, descriptions and demonstrations of being connected, working spontaneously and opening up, and beginning to process material.

### Case studies

The case studies included in this review (*n* = 38) included quantitative, qualitative, and mixed methods designs. Objectives were to explore and describe potential impacts of the arts therapies. Sample sizes ranged from one to five participants, and included mostly women. With the exception of one community sample, all other samples represented clinical populations with a diversity of presenting problems. These are listed alphabetically and included: (a) biopsychological disorders such as bipolar disorder, eating disorders, panic disorders, schizophrenia, and substance abuse, (b) chronic conditions of illness such as pain and cancer, (c) medical disorders such as Alzheimer’s disease, aphasia, brain injury, cancer, cerebral palsy, dementia, quadriplegia, stroke, (d) neurodevelopmental disorders such as autism, (e) personality disorders, (f) psychological problems, such as aggression, anxiety, depression, and emotion regulation, (g) trauma and stress problems such as adult sexual assault survivors, maltreated children, and refugees, and (h) other presenting problems such as parenting concerns and perinatal anxiety. Interventions included art therapy (i.e., drawing, painting, portraits, collage, mandala, clay, sculpting, jewelry making, mask making), music therapy, dance and movement therapy. Duration of treatment ranged from single sessions to 2 years of sessions. Frequency ranged from 4x/week to biweekly sessions. Sessions lasted 15 min to 2 h; however, frequency and duration varied and not all publications reported these details.

From an integrated arts therapies and neuroscientific perspective, case studies suggested beneficial outcomes and highlighted clinical and theoretical implications. Biophysiological instruments demonstrated increased activity in alpha and beta waves, increased activation of socioemotional areas of the brain such as limbic areas of the brain, improved cognitive functioning, and recovery of chronic propognosia, as well as decreased activity in temporo-parietal areas and occipital-temporal neuropathways associated with negativity, and decreased stress markers and trauma symptoms (Belkofer and Konopka, 2008; Fachner et al., 2019; Kang et al., 2022; Holochwost et al., 2020; Pąchalska, 2022; Pąchalska et al., 2013; Pąchalska et al., 2021; Walker et al., 2016).

Other potential outcomes from these case studies were suggested or theorized. For example, that arts therapies may lead to other neuroscientific benefits, such as balanced autonomic nervous system function, utilization of the mirror neuron system and right orbitofrontal cortex (Homann, 2010; Kang et al., 2021; Pąchalska and Góral-Półrola, 2020). Findings also suggested that arts therapies promote improvements in memory reconsolidation, visual neglect status, line bisection test performance, hemispheric integration, executive functioning, and neurophysiological, neuropsychological, and psychiatric symptoms (Hass-Cohen and Clyde Findlay, 2019; Hass-Cohen et al., 2022; Homann, 2020; Kang and Thaut, 2019; Kang et al., 2022; McNamee, 2004; Pąchalska et al., 2013; Perryman et al., 2019; Warson and Warson, 2023). Cognitive findings suggested increased self-referential and associative thinking, awareness (cognitive, affective, sensory, bodily, attachment styles, coping skills), mindfulness and mind–body connection, implicit processing and somatic perceptions, cognitive functioning, and ability to address maladaptive beliefs (Carr, 2014; Elbrecht and Antcliff, 2014; Haeyen and Hinz, 2020; Hass-Cohen and Clyde Findlay, 2009; Hass-Cohen and Clyde Findlay, 2019; Homann, 2010; Homann, 2020; Pąchalska, 2022; Pąchalska et al., 2021; Pink and Mackley, 2014; Saltzman et al., 2013).

Information from quantitative psychological assessments and qualitative data also suggested improvements in affect and behavior. Affective improvements were also reported, such as: increased hope, optimism, decision-making, agency, joy, pleasure, triumph, emotional expression (i.e., anger, other negative emotions), emotion regulation, resiliency, grief-processing, desire to live and overcome depression, as well as decreased blame, trauma symptoms, pain symptoms, perinatal anxiety, stress, and trauma symptoms (Canty, 2009; Carr, 2014; Corrado et al., 2022; Gerge et al., 2019; Guseva, 2018; Haeyen and Hinz, 2020; Hass-Cohen and Clyde Findlay, 2009; Hass-Cohen et al., 2014; Holochwost et al., 2020; Homann, 2017; Homann, 2020; Klorer, 2005; Perryman et al., 2019; Riley, 2004; Walker et al., 2016; Vaisvaser, 2019). Behaviorally, case studies reported increased self-care, verbal and non-verbal communication, ability to breastfeed, positive and trusting relational interactions, overall functioning, creativity, motivation for sobriety, boundary setting, and body autonomy, as well as decreased aggression, and anxiety and avoidance behaviors (Carr, 2014; Corrado et al., 2022; Gerge et al., 2019; Guseva, 2018; Hass-Cohen et al., 2014; Homann, 2017; Homer, 2015; Mandić-Gajić, 2018; McNamee, 2005; Netzer and Brady, 2009; Saltzman et al., 2013; Stewart, 2004) (Table 6).

**Table 6:** Case study research.

Author and year	Sample size	Population	Research design	Data analysis	Interventions	Outcomes
Belkofer and Konopka (2008)	1 (M, 29 years., White)	Adult	Quantitative	Descriptive case example, application, and comparisons. Dependent samples *t*-test.	Art-based intervention: painting or drawing (watercolor, charcoal or pencils) with no prompts, single session, 1 h.	Higher frequency bands (alpha and beta) measured by EEG were characterized by increased in brain activity after painting and drawing. Lower frequency bands were characterized showed decreases in brain activity. In the future, one may be able to define art therapy treatment based on clients’ histories as well as their baseline EEG patterns, and to use art therapy to modify or normalize the brain activity leading to improvement in their condition.
Canty (2009)	1(M)	Drug rehabilitation	Qualitative	Descriptive case example and application.	Art-based intervention: Residential community small groups, individual sessions and large groups. Each person creates an image and the group comes together to discuss.	Art therapy and group work allowed for anger to be expressed and carried by the group member which opened up dialogue between the group where similar feelings were expressed.
Carr (2014)	1 (M, 49 years)	Chronic illness, palliative care, trauma	Qualitative	Descriptive case example and application.	Art-based intervention: 14 portrait sessions, lasting between 1.5 and 2 h spread over a seven-month period, guided by art therapist.	Researcher observed that client achieved a broadening of self-referential associative thinking through the use of metaphor and symbolism in portraits. Researcher noted an increase in client’s hope and optimism, decision-making and agency. Client’s self-care (including dress and hygiene) also improved markedly after the portrait sessions.
Corrado et al. (2022)	3 (2F, 23 year White and 15 year Latina; 1 M, 10 year White)	Complex trauma, eating disorders, aggression	Qualitative	Descriptive case example and application.	Art-based intervention: Kintisugi (broken bowl project), collage, drawing.	Collage helped to identify clients desire to live and will overcome depression. Client was able to process grief/blame of mother’s death and aggression significantly reduced. Despite different modalities used in treatment, each provider expertly implemented what they thought would best address presenting problem though trauma-informed care.
Elbrecht and Antcliff (2014)	1 (F, 35 years)	Trauma	Qualitative	Descriptive case example and application.	Art-based intervention: 11 clay field sessions.	The client was able to discover the sensorimotor needs of her infant self in the clay field. The clay field became a transitional object for the client which provided her with a growing awareness of her internal and external senses, as well as new cognitive insights and an opening to rewrite her life script.
Fachner et al. (2019)	2 (2F, 72 and 65)	Emotion regulation, trauma	Mixed methods	Micro-analytic approach highlighting moments of interest, with parallel analysis of the EEG. Dependent samples *t*-test.	Music-based intervention: single session, 1.5 h, nurturing music program with two GIM therapists serving as “Guides” for each of the participants or “Travelers.”	Negative emotions occurred during moments of interest (MOI). FAA peaks of guide and traveler were similar during MOI. The temporo-parietal areas become less active during therapy.
Gerge et al. (2019)	5 (1 M, 2F, 2 unidentified)	Post-traumatic stress disorder (PTSD)	Qualitative	Narrative phenomenological analysis.	Art- and music-based intervention: Clients who had partaken in some form of long (more than a year) art-based psychotherapy were given a questionnaire by the researchers about their experience The interventions considered were: music therapy, relational art therapy in individual and group settings, and phase specific relational psychodynamic therapy with integrated art therapy.	Clients highlighted the relational aspects of the art therapies. Client reported increase their joy, pleasure, and experience of triumph as a result of their engagement in these therapies
Guseva (2018)	1 (F, Late 80s, White)	Alzheimer’s	Mixed methods	Descriptive case example, application, and comparisons; Compared Likert scale ratings of standardized measure.	Art-based intervention: Eight 30–45 min individual sessions. Sessions 1–2 mandala coloring; 3–4 collage making; 5–6 painting wooden box; 7–8 bracelet making	Increase in emotional expression and improvement in both verbal and non-verbal communication.
Haeyen and Hinz (2020)	2 (2F; 57 years., White; 58 years)	Personality disorders	Qualitative	Descriptive case example and application.	Art-based intervention: One session guided by art therapist (drawing with markers, clay sculpting). Data was gathered from the first 15 min of the session.	The first 15 min of an art therapy session provided information about clients’ attachment styles and emotional regulation strategies. The first 15 min of art therapy demonstrated clients’ favored components of the expressive therapies continuum (ETC).
Hass-Cohen and Clyde Findlay (2009)	1 (F, 64, White)	Chronic pain	Qualitative	Descriptive case example and application.	Art-based intervention: Art therapy relational neurobiology (ATR-N) brief assessment protocol (drawings, autobiographical portrait), guided by art therapist, two interviews lasting 2 h each.	Client was able to imagine how the exploration of the sensory, emotional, cognitive, and social aspects of pain can give hope for less painful possibilities and reveal coping skills. The study suggests preliminary exploratory support for an ATR-N protocol assessment of capacity for change as well as for treatment purposes of chronic pain symptom reduction and pain management.
Hass-Cohen and Clyde Findlay (2019)	1 (F, Veteran)	Trauma, depression, anxiety, panic	Qualitative	Descriptive case example and application.	Art-based intervention: Four-drawing protocol which can be completed in one or two sessions, guided by art therapist.	Protocol promotes memory reconsolidation through the targeting of explicit and implicit memories where the arts compete visually and vividly with old memories.
Hass-Cohen et al. (2014)	1 (1F)	PTSD	Quantitative	Descriptive case example, application, and comparisons; Compared Likert scale ratings of standardized measures.	Art-based intervention: Check-change drawing protocol, single session, guided by art therapist.	Decreased anxiety and avoidance behaviors and improved resiliency from pre-test to pos*t*-test.
Hass-Cohen et al. (2022)	4 (4F), between ages of 20–40, all White	Chronic pain	Qualitative (semi-structured in-depth interviews)	Inductive analysis for common themes.	Art-based intervention: Three- and four-drawing protocol, single session, 1-h.	Positive outcomes included less physiological pain, psychological and skills-based changes in pain, and more access to coping resources. Art therapy-based memory reconsolidation change factors were also identified, including creative processes and protocol sequencing.
Holochwost et al. (2020)	4 parent–child dyads (mothers 34–42 years and children 3.5–4.5 years; two White, one Black, one multiracial)		Mixed methods	Thematic analysis of interviews; Tau-U statistics	Music-based intervention: Active Music Engagement (AME), two 45-min sessions per week, completing 7 to 8 sessions across 4 weeks	AME was found to be feasible, salivary cortisol data collection methods were seen as a burden. Interview data suggested perceived benefits in relief and decreased stress for children and caregivers during and after AME sessions, and that families used AME outside of therapy sessions. Salivary cortisol data showed a pattern of increased distress due to not knowing how the medical treatment would go, and then decline in stress later attributed to AME.
Homann (2017)	1 (F, it was reported that the client was a young, pregnant refugee)	Perinatal	Qualitative	Descriptive case example and application.	Dance- and movement-based intervention: initial sessions began with breathing exercises, later sessions involved dancing using movement metaphors. After the baby was born, mother danced with the baby during sessions and did movement to support breastfeeding.	The client started to feel less anxious regarding her pregnancy and the birth of her first child and was able to breastfeed. The client was able to engage her mind and body and remember that her body belongs to her.
Homann (2010)	Clinical Group Vignette, 2 (F); Individual Case Vignettes, 2 (1 M, 1F); Group Vignette, 2 (1F, 1 unreported)	Interpersonal relations, cancer, emotional distress	Qualitative	Descriptive case example and application.	Dance- and movement-based intervention: Movement experience in pairs.	The movement therapy allowed clients to elicit more awareness, implicit processing and mind/body connection. Individual movement therapy utilized implicit somatic dimension of perception. Interaction with the therapist through embodied movement activates the mirror neuron system and thus the limbic areas, autonomous nervous system and right orbitofrontal cortex, which all leads to more mindfulness.
Homann (2020)	5 (4F, 1 M)	Refugees, trauma	Qualitative	Descriptive case example and application.	Dance- and movement-based intervention: Embodied interoception.	DMT allowed for a perceptual shift which thus led to greater body and emotional awareness. Assisted in treating traumatic response to pregnancy. DMT helped a patient with trauma and cognitive impairment to develop trust in new relationships. DMT also created an experiential process that assisted in processing grief and the reintegration of memories.
Homer (2015)	1 (F, 25, White)	Personality disorder and bipolar disorder	Qualitative	Descriptive case example and application.	Art-based intervention: Collaborative fabric collage, guided by art therapist, took place over several months.	Client reported progress in determining an appropriate level of attachment and boundary setting. A year after therapy concluded, client contacted researcher and reported that her new therapist had reassessed her diagnosis and determined that she no longer met the criteria for borderline personality disorder. Suggests that a biologically respectful treatment that offers relational, relevant, repetitive, rewarding, and rhythmic activities may help ameliorate trauma/
Kang et al. (2022)	3 (3F)	Quadriplegia; cerebral palsy	Quantitative	Descriptive case example, application, and comparisons. Two-way mixed ANOVA.	Music-based intervention: Four sessions, facilitated by music therapist where they played guitar and sang participants’ favorite songs. Four storytelling sessions where therapist read the participants favorite books. All sessions included an engagement aspect, such as including the participant in the song or story.	Significantly increased brain activity for music rather than storytelling in the socio-emotional areas.
Kang and Thaut (2019)	2 (1F, 62; 1 M, 69)	Stroke	Quantitative	Descriptive case example, application, and comparisons. Dependent samples *t*-test and Wilcoxon signed rank tests.	Music-based intervention: Six individual sessions, 2x/week, 30 min each, over a period of 3 weeks. Musical Neglect Training (MNT) sessions. Guided by experimenter.	Both participants showed improvements in visual neglect status (Albert’s test). Both participants showed small but insignificant improvements on the line bisection test. Overall, study presents the positive potential of MNT for patients with chronic persistent visual neglect.
Kellman (1996)	4 (2 M, 2F)	Autism	Qualitative	Descriptive case example and application.	Art-based intervention: drawing, painting.	There is a unique triadic relationship between the art, artist, and pre-attentive vision among artists with autism, and that insight into this relationship could provide a basis for understanding the special physiological predispositions of gifted artists. The use of unusual characteristics and the non-conceptual nature of these artists’ drawings were indicative of their reliance on pre-attentive processes in their artwork, which offered a clearer view of the visual world because of their apparent diminished ability to conceptualize.
Klorer (2005)	2 (1 M, 1F)	Severely maltreated children	Qualitative	Descriptive case example and application.	Art-based intervention: No structured intervention, just creative therapies of child’s choosing.	Maltreated children able to express themselves freely through art in ways they were unable to with words.
Mandić-Gajić (2018)	2 (2 M, 31 and 36)	Alcoholics	Qualitative	Descriptive case example and application.	Art-based intervention: Free association drawings with group analysis.	Analysis of drawings allowed one participant to notice consequences and impairments due to alcoholism and motivated continued sobriety (ex: distorted lines and perspectives) other participant responded with anger and does not appear to have completed final drawing.
McNamee (2004)	1 (F, mid 20s)	Depression, anxiety, panic attacks	Qualitative	Descriptive case example and application.	Art-based intervention: Annotated scribble drawings and verbal discourse about images in drawings, guided by art therapist. 14 weeks of treatment.	Scribble drawings provided client with a means for identifying her own truths. The narrative that client produced in response to the images served to integrate her right-hemisphere experiences with left hemisphere understanding.
McNamee (2005)	1 (F, 22)	Perinatal trauma (losses and miscarriage)	Qualitative	Descriptive case example and application.	Art-based intervention: Bilateral art drawing protocol. Family art therapy done over 24 sessions (12 individual sessions, 10 couples sessions, and 2 family sessions).	Self-reported improvement to level of functioning, reduction of complaints regarding presenting complaint and improved relational interactions.
Netzer and Brady (2009)	2 (1F, 1 M)	Parenting	Qualitative	Descriptive case example and application.	Art-based intervention: Case 1- Abstract drawing. Case 2- Non-directed drawing.	Using an open-minded approach, not trying to know or question the artmaking process but ground its meaning to real life helps to understand the child relationship. The authors develop a metaphor of building a house and how the process of compromise and mutuality helps strengthen the relationship.
Pąchalska et al. (2013)	1 (M, 68, White)	Schizophrenia; traumatic brain injury (TBI)	Quantitative standardized neuropsychological testing and event-related potentials (ERPs)	Descriptive case example, application, and comparisons.	Art-based intervention: Painting post neurotherapy, 20 sessions of relative beta training (enhancing beta activity recorded over the frontal electrodes). Each session included approximately 20 min of neurofeedback training.	From pretest to posttest, there was marked improvement of neurophysiological, neuropsychological, and psychiatric symptoms, as well as executive dysfunction and behavioral disorders.
Pąchalska and Góral-Półrola (2020)	2 (1 M, 1 unreported)	Aphasia	Mixed methods	Descriptive case example, application, and comparisons. Case examples from other studies.	Art-based intervention: Drawing and painting.	A lack of negative components in the occipital-temporal region of the brain is seen in those with aphasia when compared to healthy participants. The article further goes on to discuss art therapy methods and approaches.
Pąchalska et al. (2021)	1 (M, 23)	Prosopagnosia; stroke	Mixed methods	Descriptive case example, application, and comparisons	Art-based intervention: Drawing, portraits. 1x/week for 10 weeks.	tDCS and art therapy assisted in the recovery of chronic prosopagnosia.
Pąchalska (2022)	1 (M)	TBI	Mixed methods	Thematic analysis of the art. Comparisons to quantitative findings from other studies.	Art-based intervention: Self-portraits, drawing, painting. (Clinical material; Therapy of Symbolic Thought). Four times per week for 2 years.	Using art therapy can be particularly beneficial in treating TBI and rebuilding creative abilities. There were significant improvements in cognitive functions after 1 year of treatment, but indicators of sadness and loneliness still remained.
Perryman et al. (2019)	1 (F, 60)	Trauma, depression	Qualitative	Descriptive case example, application, and analysis	Art- and mindfulness-based intervention: Mindfulness, finger paint, sand tray, and journaling activities (Occurred as a part of regular therapy)	Targeting both the right and left hemispheres with different activities, the client was able to work through her trauma with the clinician. At the end of treatment (over 4 months) she reported feelings of hope and being back to “normal.”
Pink and Mackley (2014)	2F	Adults	Qualitative	Photo and video ethnography	Photo- and Video-based intervention: Photographing and videoing re-enactments of everyday activities (i.e., laundry).	Personal identity, sensory knowledge, and embodied knowledge are shown through video ethnography as participants embed these aspects into their movements and dialogue as they progress through the task.
Riley (2004)	3 (2F, 1 M)	Adults	Qualitative	Descriptive case example, application, and comparisons	Art-based intervention: Psychoeducation about neuroscience in the art therapy session (i.e., explaining process like left versus right brain, neurons, and synaptic pruning). All artmaking process were unguided drawings but were based on psychoeducation conversations.	Incorporating neuroscience psychoeducation can benefit the art therapy session as new information can help provide context for the clients’ experiences and inform the content of their art to exemplify meaningful change or emotion.
Saltzman et al. (2013)	3 (1 M, 14, African American; 2F, 23, Russian and African and 13, African American)	Sexual assault and assault	Qualitative	Descriptive case example, application, and comparisons	Art-based intervention: Adlerian art therapy. Mask making, drawing. Intervention was administered as part of individual and/or group treatment of trauma (varied by case).	Case examples demonstrated how Adlerian art therapy can build a relational connection between the survivor and others while also addressing maladaptive beliefs.
Stewart (2004)	4 (3F, 1 M)	Dementia	Qualitative	Descriptive case example, application, and comparisons	Art-based intervention: Drawing, painting, clay, collage, chalk.	Artmaking allowed for greater self-expression and creativity in elderly adults with advanced dementia, also highlighted overlearned abilities.
Vaisvaser (2019)	3 M (6 years old, 5.5 years old, 5.5 years old)	Youth with autism	Qualitative	Descriptive case example, application, and comparisons	Art-, music-, dance- and movement-based interventions: 30 weekly, 45-min group sessions, comprising 4 phases: opening song, drawing on paper, free movement with stretch bands, and closing song. Co-led by occupational therapist and a dance movement therapist.	The group provided a defined yet flexible space for spontaneous explorations and responsiveness. Motivation to connect and be seen was revealed realized among all three children participating in the group. Moments of “state sharing” emerged in the group, dialogues of shared sounds and body movements.
Walker et al. (2016)	1 (M, active duty service member in his 50s)	TBI; PTSD	Mixed methods	Descriptive case example, application, and comparisons	Art-based intervention: Mask-making, collages, paintings, etc. and acupuncture. Guided by art therapist and acupuncture physician. 14 sessions, lasting 1–2 h.	Patient experienced alleviation of persistent symptoms of intrusive images and traumatic memories.
Warson and Warson (2023)	1 (F, 13, Latina and White)	Trauma	Mixed methods	Descriptive case example, application, and comparisons	Art-based intervention: Airdrawing, Bilateral drawing (chalk, pastels), incorporating EMDR.	Bilateral movement and artmaking across the midline enhanced hemispheric integration.

### Summary

The results are summarized according to modalities, methodologies, instruments, and measures, as well as population and context, across all articles included in this scoping review.

#### Modalities

Across all research methodologies, the main modalities included in the articles were: art therapy (*n* = 64), music therapy (*n* = 14), dance movement therapy (*n* = 10), neuroaesthetics (*n* = 4), drama therapy (*n* = 3), and phototherapy (*n* = 1). Art therapy was the most common modality, and several different types of interventions were reported, including clay, collage, coloring, digital art, drawing, painting, and sculpting. Many articles covered multiple modalities of arts therapies within one publication (*n* = 34).

#### Methodologies

The most common publications were case studies (almost half), and the second most common were quantitative studies (about a third). There were about a tenth of mixed methods studies, and few review and qualitative articles. This distribution of integrated arts therapies and neuroscience articles is broad but also imbalanced.

A methodological strength was the number of quantitative publications that included randomization of participants to control or comparison groups, and the use of high-tech instruments and standardized measures. Some methodological weaknesses included small sample sizes, more women than men participants, simple statistical analyses with lower power and higher likelihood of error, and a lack of consistently reporting details about the intervention (i.e., frequency and duration).

Certain modalities were only studied using certain methodologies. For example, neuroaesthetics and photo therapy were only included in quantitative research studies, and drama- and performance art-based therapies were included in review, quantitative, and mixed methods research. However, art therapy and music therapy were utilized in all methodologies.

#### Instruments and measures

Biophysiological instruments, neuropsychological and psychological standardized assessments, cognitive, perceptual, and performance-based tasks, qualitative interviews, and arts-based assessments were used across all reviewed studies. Standardized psychological measures were very common across studies that collected quantitative data. Interviews were very common across studies that included qualitative data. Of the biophysiological instruments, EEG was the most common, then fMRI.

#### Population and context

The context in which most studies took place varied, including individual therapy, hospitals, museums, group therapy, care facilities/nursing homes, rehabilitation programs, schools, research labs, and other generic settings. Medical populations were the most common, and included neurological problems (including Alzheimer’s, aphasia, dementia, neurological impairment, Parkinson’s, prosopagnosia, stroke, and TBI; *n* = 25), pain or injury (including chronic pain, multiple sclerosis, physical rehabilitation from injury, and pain management; *n* = 5), chronic illness (including cancer, chronic illness; *n* = 4). The second most common population studied were general community adult samples (*n* = 20), and trauma (including resilience, trauma, grief, stress, and Veteran populations; *n* = 19). A minority of the studies focused on psychological functioning (including anxiety, depression, emotion regulation; *n* = 5), neurodevelopmental problems (including autism and developmental disabilities; *n* = 5), schizophrenia (*n* = 2), substance use (*n* = 2), parent–child dynamics (*n* = 1), and art therapy providers (*n* = 1).

## Discussion

The discussion of the findings was organized across the review, quantitative, mixed methods, qualitative, and case studies. The focus was on found communalities and unique aspects. The purpose was to map the state of the field and inform future directions.

### Strengths and limitations of review articles

There were four review articles, with one meta-analysis from 2020. Most of the review articles focused on medical presenting problems, aside from the meta-analysis, which included publications focused on art therapy interventions with non-clinical adult samples. Findings were broad across diverse populations, problems, and functions, for example: cognitive and motor function. The main psychological findings were related to mood disorders, quality of life, and social outcomes. There was no consistent arts therapies and neuroscience theme, thread, or focus.

A strength of these review articles is that they were relatively current to the publication of this scoping review. A limitation was that empirically strong meta-analysis focused on art therapy interventions. This finding suggested that review articles on integrated arts therapies (with a diversity of modalities) and neuroscience are limited; thus, supporting the need for this scoping review, meta-analyses, and other review forms in the future. This paper provides an extensive review that maps out the current available research through charting and narrative summaries. The current scoping review of 84 articles, far surpasses the largest previous review of 46 articles (Malik, 2022; Tramontano et al., 2021); thus, ameliorating the gap that most reviews have been brief and limited (Oliva et al., 2023).

### Contributions of quantitative research

Quantitative publications were the second most common publication, which underscores the growing importance of empirically supported integrated arts therapies and neuroscience research. A strength was that many of the articles were within the past 10 years. Of the quantitative publications, about half were focused on neurological disorders and about a third were about non-clinical community adult samples. Over half of the quantitative publications utilized art therapy, while other modalities included music, dance, neuroaesthetics, performance, and photo therapies. These results demonstrated that all the arts therapies modalities included in this review have some empirical support, and that the most empirically supported modality was art therapy. There were slightly more female and white participants in these studies, which is common in social science research. However, future researchers should make further efforts to fill in the gaps with more diverse samples with more varied clinical presenting problems.

A strength found by this current review of the quantitative studies was that over three fourths of the studies included a control or comparison group, and half of the studies included random assignment to groups. However, the statistical analyses chosen for these research designs were somewhat weak due to low power from small sample sizes, or less than ideal selection of statistical tests (i.e., using multiple dependent samples *t*-tests instead of one repeated measures ANOVA, or using dependent samples *t*-tests with a small sample instead of using Wilcoxon signed ranks test); thus, leading to increased risks of error and threats to validity and generalizability of the results. Results suggested improvements in cognitive function, sensory function, motor function, and executive function, as well as decreases in stress, anxiety, depression, pain, OCD symptoms, and increases in quality of life, relationship quality, memory, positivity, and creativity. There were no shared integrated arts therapies and neuroscience threads across all quantitative publications. Articles selected an arts therapies and neuroscience focus without a rationale tying it to previous research. This finding supported one of the gaps identified earlier, that integrated arts therapies and neuroscience research has addressed some but not all neuropsychological psychological domains (Czamanski-Cohen and Weihs, 2016; Malik, 2022; Strang, 2024). A mapping of arts therapies and neuroscience foci is needed to systematically provide focused direction for future research; thus, highlighting the importance of this publication in providing a step toward this goal.

### Mixed methods research themes

Mixed methods research studies topics focused on Parkinson’s, cancer, trauma, and community non-clinical samples. All but one study were focused on adults, with an even distribution of medical, psychological, and non-clinical populations. Demographics were evenly balanced for male and female participants. These research studies predominantly included art-based interventions, aside from one focused on drama therapy. Interventions varied in frequency and duration. Mixed methods designs were predominantly pretest-posttest designs with thematic analyses of participant narrative self-reports. Most sample sizes were relatively small for the statistical tests that were used; therefore, limiting generalizability of the results. Results suggested improvements in positive affect, emotional awareness and processing, and decreases in phobias, OCD, and trauma, as well as negative affect and emotional and somatic symptoms. Findings supported these results with perceived and self-reported improvements in positive affect and emotional expression, as well as highlighting meaning making experiences. Relative to quantitative and case studies, only about a tenth of the total studies were mixed methods designs. The rationale for a mixed methods research did not seem to be grounded in the aims of arts therapies and neuroscience integration, nor for specific populations. Again, shared arts therapies and neuroscience themes or approaches were lacking, thus, constraining future research directions, and further contributing to the gaps (King and Strang, 2024).

### Paucity of qualitative research

There were only a few qualitative studies. Participants included art therapy clinicians and graduate students, adults with chronic pain, veterans with PTSD, and community samples of adults. Most participants in these studies were female, and sample sizes ranged broadly from small to large. Qualitative studies focused on single session, art therapy interventions; however, duration was not well described. Findings suggested that qualitative studies used thematic analyses to describe neuroscientific aspects related to attachment, safety, and trauma processing. Qualitative outcomes related to cognitive, affective, and social functioning related to pain were described. These articles contributed to theoretical understandings of the connections between arts therapies and neuroscience (Hass-Cohen and Carr, 2008; Czamanski-Cohen and Weihs, 2016). However, when compared to other types of publications, additional qualitative publications are needed to support the development of constructs needed to inform quantitative research.

### Prevalence of case study research

Case studies were the most common type of publication and included single participants and small samples of a diversity of clinical problems, which ranged from non-clinical community samples to severe mental health and medical disorders, including: biopsychological disorders, chronic illness, medical disorders, neurodevelopmental disorders, personality disorders, psychological problems, trauma, and social problems. A majority of the participants were adult women. Case studies focused on arts therapies, music therapy, and dance movement therapy with varied frequency and duration. Case studies included quantitative, qualitative, and mixed methods data. Including pre- and pos*t*-test findings was a strength of the case studies. Outcomes were limited in that not all studies reported the details on the intervention. Data was gathered from biophysiological instruments, psychological assessments, self-report surveys, interviews, and artwork. The use of biophysiological instruments was a strength of case studies and contributed to closing the gap between theory and empirical backing (Malik, 2022). Case study publications presented the neuropsychological impacts of arts therapies, as well as suggested neurological evidence for the integration of arts psychotherapies and neuroscience. Outcomes suggested improvements in brain activity and integration, cognitive functioning, memory reconsolidation, psychological symptoms, affect, and behavior. The current review of these case studies incorporating biopsychosocial instruments could be used for the development across arts therapies and neuroscience research (Strang, 2024).

### Implications

Based on these findings, clinicians and researchers alike may be able to glean a better idea of the state of the field, as well as interpret implications for limitations and suggested future directions in these topic areas. For example, the most frequently researched modality of all the publications on integrated arts therapies and neuroscience, was art therapy. This suggests that its connections to neuroscience have been considered and studied the most, which may lead to it being more understood and accepted in the field; however, further research and documented clinical work needs to be conducted on other modalities to be able to decipher what is most beneficial for diverse clients and presenting concerns. Many studies were focused on medical issues, perhaps suggesting that most research in this integrated field relies upon medical art therapists who have access to populations with medical concerns, and the ability to collect neuroscience data using biophysiological measures. This also suggests that art therapy is more accepted and deemed as feasible in a hospital or neuroscience research setting than other areas of psychological research. The most frequently reported methodology incorporating arts therapies and neuroscience were case studies; thus, suggesting that to this point, it has been the most feasible way to integrate these topic areas. Further research with a range of modalities, especially those with more high-quality design and empirical strength, are needed.

### Limitations

There were limitations specific to different methodologies. For example, the rationale for the quantitative studies seemed to be idiosyncratic to the researchers’ interests, and do not follow an overarching trend or articulated rationale for the integration of neuroscience and arts therapies. The purpose of the qualitative outcomes seemed to be more in support of building theory, with the exception of one publication, which was a follow-up to a quantitative study. It is not clear how the qualitative studies may contribute to other future evidence-based or quantitative empirical studies. A limitation of the case studies was that some theorized outcomes were often based on one or few participants.

More generally, a limitation in this field of study is that most arts therapists may not have access to biophysiological instruments, and therefore are hypothesizing about changes and processes in the brain based on prior research and theories without being able to measure them in research studies. This is an area of future transdisciplinary research that would benefit this integrated field (Malik, 2022). It is suggested that art therapy researchers collaborate with neurobiological researchers to be able to have support in accessing biophysiological instruments and interpreting data collected.

One of the main aims of arts therapies and neuroscience is to identify neural pathways responsible for mechanisms of change in arts therapies, to understand neural processes and support the effectiveness of arts therapies (Strang, 2024). However, the general scatter of methodologies and modalities included in these publications is what makes it difficult on how the evidence can clearly inform pathways in the field based on how systems interact in this integrated arts therapies and neuroscience field. This heterogeneity compromises the comparability of the results, thus, limiting the generalizability of findings and ability to differentiate effectiveness based on methodology and modality. It is suggested that more researchers used mixed methods to be able to speak to quantitative and qualitative effects of operationalized interventions, and to target specific modalities and presenting problems. This is still emergent and hopefully will be clarified as the field continues to develop and grow in its supportive evidence base.

Any publications on integrated arts therapies and neuroscience may have been missed, as more may have been published since this review was conducted in 2024. However, this review was able to include more publications than previous reviews, specifically 25 quantitative studies, which more than doubles what had been found only a few years earlier (Malik, 2022); thus, suggesting the rapid proliferation of publications in this integrated field. Overarching limitations across all publications included a lack in methodological strength of the research design, and statistical analyses. Most articles were theoretical, meaning they described how neuroscience may play a role in arts therapies. While these articles are helpful in understanding possible connections in this field and may inspire areas of future research, they are limited in that it is difficult to assess accuracy of these theories until further research is conducted. In addition, the collection of integrated arts therapies and neuroscience research lacks directionality in approach, as there are too many differences across publications and a lack of clear definitions, which makes it difficult to determine neural correlates to certain modalities and outcomes. Most research is exploratory and dependent on researcher assumptions, expertise, and accessibility to certain instruments and populations.

### Future directions

Future directions for each methodology and general recommendations are considered. For purposes of meta-analysis, additional high quality, quantitative research on multiple forms of arts therapies and more diverse populations and specific presenting problems must be conducted to have more publications that are eligible to be included. For mixed methods research, the recommendation is to continue to engage in such methodologies, as there were less of these publications, and no Delphi studies were noted. Mixed methods studies have the potential to better explain quantitative data, fine-tune interventions, and could be useful for program design. It may behoove the field to consider engaging in such methodologies that include feedback from experts in the field.

For general recommendations, the use of multiple modalities in almost half of the publications allowed for understanding of overarching neuroscientific benefits of arts therapies as well as benefits that were unique to individual modalities. Further research may solidify understanding in the field about what specific neuroscientific processes and benefits are present for each individual modality. Standardization of methodologies, instruments and measures, and use and implementation of modalities with diverse populations, as well as focus across researchers will happen in time in order to support the further development of this integrated field and will support attempts to contribute to theories and research of arts therapies, effectively.

## Conclusion

The main objectives of this scoping review were met in that there is now an existing map of the state of the field of integrated arts therapies and neuroscience research. This scoping review provides a comprehensive body of knowledge based on the review, quantitative, qualitative, and case study publications on integrated arts therapies and neuroscience. A review of theoretical publications further supporting the understanding of integration arts therapies and neuroscience from the current authors will be forthcoming. This scoping review is unique in that it included multiple modalities of arts therapies and diverse psychological topics. This review summarized and synthesized populations, settings, presenting problems, instruments, measures, and outcomes included in integrated arts therapies and neuroscience research. While this scoping review was able to summarize and synthesize the state of the field of integrated arts therapies and neuroscience, it is still too early to be able to compare and contrast results and findings or make more solid conclusions. Considering the variability of the reviewed publications, it is recommended that there is greater methodological alignment among future studies. For example, if there were more methodologically strong quantitative studies with large sample sizes and advanced statistical analyses, this would allow for more meta-analyses to be conducted to better inform the effect of arts therapies. This scoping review is of utmost importance as it provided implications for the field and made suggestions for future directions, which can be of value for both researchers and clinicians.

## Data Availability

The original contributions presented in the study are included in the article/Supplementary material, further inquiries can be directed to the corresponding author.

## References

[ref1] Abbing A. C. Baars E. W. Van Haastrecht O. Ponstein A. S. (2019). Acceptance of anxiety through art therapy: a case report exploring how anthroposophic art therapy addresses emotion regulation and executive functioning. Case Rep. Psychiatry 2019, 1–13. doi: 10.1155/2019/4875381, PMID: 32082678 PMC6949689

[ref2] American Psychological Association (2025). APA dictionary of psychology. Washington, DC: APA.

[ref3] Bar A. Czamanski-Cohen J. Federman J. D. (2021). I feel like I am flying and full of life: contemporary dance for Parkinson's patients. Front. Psychol. 12:623721. doi: 10.3389/fpsyg.2021.623721, PMID: 34290638 PMC8287013

[ref4] Bastepe-Gray S. Wainwright L. Lanham D. C. Gomez G. Kim J. S. Forshee Z. . (2022). GuitarPD: a randomized pilot study on the impact of nontraditional guitar instruction on functional movement and well-being in Parkinson's disease. Parkinson's Disease 2022:1061045. doi: 10.1155/2022/1061045, PMID: 35795456 PMC9252755

[ref5] Belkofer C. M. Konopka L. M. (2008). Conducting art therapy research using quantitative EEG measures. Art Ther. 25, 56–63. doi: 10.1080/07421656.2008.10129412

[ref6] Belkofer C. M. Van Hecke A. V. Konopka L. M. (2014). Effects of drawing on alpha activity: a quantitative EEG study with implications for art therapy. Art Ther. 31, 61–68. doi: 10.1080/07421656.2014.903821

[ref7] Bolwerk A. Mack-Andrick J. Lang F. R. Dörfler A. Maihöfner C. (2014). How art changes your brain: differential effects of visual art production and cognitive art evaluation on functional brain connectivity. PLoS One 9:e101035. doi: 10.1371/journal.pone.0101035, PMID: 24983951 PMC4077746

[ref8] Canty J. (2009). The key to being in the right mind. Int. J. Art Ther. 14, 11–16. doi: 10.1080/17454830903006083

[ref9] Carr S. M. D. (2014). Revisioning self-identity: the role of portraits, neuroscience and the art therapist’s ‘third hand.’. Int. J. Art Ther. 19, 54–70. doi: 10.1080/17454832.2014.906476

[ref10] Choi A.-N. Lee M. S. Cheong K.-J. Lee J.-S. (2009). Effects of group music intervention on behavioral and psychological symptoms in patients with dementia: a pilot-controlled trial. Int. J. Neurosci. 119, 471–481. doi: 10.1080/00207450802328136, PMID: 19229716

[ref11] Corbett B. A. Ioannou S. Key A. P. Coke C. Muscatello R. Vandekar S. . (2019). Treatment effects in social cognition and behavior following a theater-based intervention for youth with autism. Dev. Neuropsychol. 44, 481–494. doi: 10.1080/87565641.2019.1676244, PMID: 31589087 PMC6818093

[ref12] Corrado M. Wolf D. Bills L. (2022). Trauma triptych: inviting cross-disciplinary collaboration in art therapy, social work, and psychiatry. Int. J. Art Ther. 27, 169–181. doi: 10.1080/17454832.2022.2123011, PMID: 40101104

[ref13] Costa A. M. Alves R. Castro S. L. Vicente S. Silva S. (2020). Exploring the effects of guided vs. unguided art therapy methods. Behav. Sci. 10:65. doi: 10.3390/bs10030065, PMID: 32156095 PMC7139608

[ref15] Cucca A. Di Rocco A. Acosta I. Beheshti M. Berberian M. Bertisch H. C. . (2021). Art therapy for Parkinson’s disease. Parkinsonism Relat. Disord. 84, 148–154. doi: 10.1016/j.parkreldis.2021.01.013 33526323

[ref16] Czamanski-Cohen J. Weihs K. L. (2016). The bodymind model: a platform for studying the mechanisms of change induced by art therapy. Arts Psychother. 51, 63–71. doi: 10.1016/j.aip.2016.08.006, PMID: 27777492 PMC5074079

[ref17] Czamanski-Cohen J. Wiley J. F. Sela N. Caspi O. Weihs K. (2019). The role of emotional processing in art therapy (REPAT) for breast cancer patients. J. Psychosoc. Oncol. 37, 586–598. doi: 10.1080/07347332.2019.1590491, PMID: 30929590

[ref18] De Bartolo D. Morone G. Giordani G. Antonucci G. Russo V. Fusco A. . (2020). Effect of different music genres on gait patterns in Parkinson's disease. Neurol. Sci. Off. J. Italian Neurol. Soc. Clin. Neurophysiol. 41, 575–582. doi: 10.1007/s10072-019-04127-4, PMID: 31713758

[ref19] Elbrecht C. Antcliff L. R. (2014). Being touched through touch. Trauma treatment through haptic perception at the clay field: a sensorimotor art therapy. Int. J. Art Ther. 19, 19–30. doi: 10.1080/17454832.2014.880932, PMID: 40101104

[ref20] Elkis-Abuhoff D. L. Gaydos M. (2018). Medical art therapy research moves forward: a review of clay manipulation with Parkinson’s disease. Art Ther. 35, 68–76. doi: 10.1080/07421656.2018.1483162

[ref21] Elkis-Abuhoff D. L. Gaydos M. Poland E. Sena S. (2022). Exploring the effects of nature-based art therapy on happiness and life satisfaction. Arts Psychother. 81:101966. doi: 10.1016/j.aip.2022.101966

[ref22] Elkis-Abuhoff D. Goldblatt R. B. Gaydos M. Convery C. (2013). A pilot study to determine the psychological effects of manipulation of therapeutic art forms among patients with Parkinson's disease. Int. J. Art Ther. 18, 113–121. doi: 10.1080/17454832.2013.797481

[ref23] Elkis-Abuhoff D. L. Goldblatt R. B. Gaydos M. Corrato S. (2008). Effects of clay manipulation on somatic dysfunction and emotional distress in patients with Parkinson’s disease. Art Ther. 25, 122–128. doi: 10.1080/07421656.2008.10129596, PMID: 40101104

[ref24] Ettinger T. Berberian M. Acosta I. Cucca A. Feigin A. Genovese D. . (2023). Art therapy as a comprehensive complementary treatment for parkinson’s disease. Front. Hum. Neurosci. 17:1110531. doi: 10.3389/fnhum.2023.1110531, PMID: 37250693 PMC10215005

[ref25] Fachner J. C. Clemens M. Denise G. Inge N. P. Gro T. Gerhard T. . (2019). “Telling me not to worry…” Hyperscanning and neural dynamics of emotion processing during guided imagery and music. Front. Psychol. 10:1561. doi: 10.3389/fpsyg.2019.01561, PMID: 31402880 PMC6673756

[ref26] Fisher M. Kuhlmann N. Moulin H. Sack J. Lazuk T. Gold I. (2020). Effects of improvisational dance movement therapy on balance and cognition in Parkinson’s disease. Phys. Occup. Ther. Geriatr. 38, 385–399. doi: 10.1080/02703181.2020.1765943, PMID: 40101104

[ref27] Gerge A. (2017). What does safety look like? Implications for a preliminary resource and regulation-focused art therapy assessment tool. Arts Psychother. 54, 105–121. doi: 10.1016/j.aip.2017.04.003

[ref28] Gerge A. Hawes J. Eklöf L. Pedersen I. N. (2019). Proposed mechanisms of change in arts-based psychotherapies. Voices: World Forum Music Ther. 19:31. doi: 10.15845/voices.v19i2.2564

[ref29] Goldblatt R. Elkis-Abuhoff D. Gaydos M. Rose S. Casey S. (2011). Unlocking conflict through creative expression. Arts Psychother. 38, 104–108. doi: 10.1016/j.aip.2010.12.006, PMID: 40182660

[ref30] Griffith F. J. Bingman V. P. (2020). Drawing on the brain: an ALE meta-analysis of functional brain activation during drawing. Arts Psychother. 71:101690. doi: 10.1016/j.aip.2020.101690

[ref31] Guseva E. (2018). Bridging art therapy and neuroscience: emotional expression and communication in an individual with late-stage Alzheimer’s. Art Ther. 35, 138–147. doi: 10.1080/07421656.2018.1524260

[ref32] Haeyen S. Hinz L. (2020). The first 15 min in art therapy: painting a picture from the past. Arts Psychother. 71:101718. doi: 10.1016/j.aip.2020.101718

[ref33] Haiblum-Itskovitch S. Czamanski-Cohen J. Galili G. (2018). Emotional response and changes in heart rate variability following art-making with three different art materials. Front. Psychol. 9:968. doi: 10.3389/fpsyg.2018.00968, PMID: 29967587 PMC6015920

[ref34] Hass-Cohen N. Bokoch R. Clyde Findlay J. Banford Witting A. (2018). A four-drawing art therapy trauma and resiliency protocol study. Arts Psychother. 61, 44–56. doi: 10.1016/j.aip.2018.02.003, PMID: 40182660

[ref35] Hass-Cohen N. Bokoch R. Goodman K. Conover K. J. (2021). Art therapy drawing protocols for chronic pain: quantitative results from a mixed method pilot study. Arts Psychother. 73:101749. doi: 10.1016/j.aip.2020.101749

[ref36] Hass-Cohen N. Bokoch R. McAnuff J. (2022). A year later: the pain protocol study findings and memory reconsolidation factors. Arts Psychother. 80:101949. doi: 10.1016/j.aip.2022.101949, PMID: 40182660

[ref37] Hass-Cohen N. Clyde Findlay J. (2009). Pain, attachment, and meaning making: report on an art therapy relational neuroscience assessment protocol. Arts Psychother. 36, 175–184. doi: 10.1016/j.aip.2009.02.003

[ref38] Hass-Cohen N. Clyde Findlay J. (2019). The art therapy relational neuroscience and memory reconsolidation four drawing protocol. Arts Psychother. 63, 51–59. doi: 10.1016/j.aip.2019.03.002

[ref39] Hass-Cohen N. Clyde Findlay J. Carr R. Vanderlan J. (2014). “Check, change what you need to change and/or keep what you want”: an art therapy neurobiological-based trauma protocol. Art Ther. 31, 69–78. doi: 10.1080/07421656.2014.903825, PMID: 40101104

[ref9004] Hass-Cohen N. Carr R. (2008). Art Therapy and Clinical Neuroscience. London: Jessica Kingsley Publishers.

[ref9001] Hass-Cohen N. Clyde Findlay J. (2015). Art Therapy & the Neuroscience of Relationships, Creativity, & Resiliency: Skills and Practices. First edition. New York: W.W. Norton & Company.

[ref40] Hass-Cohen N. Kim S. K. Mangassarian S. (2015). Art mediated intra-interpersonal touch and space: Korean art therapy graduate students’ cultural perspectives on sharing attachment-based cloth albums. Arts Psychother. 46, 1–8. doi: 10.1016/j.aip.2015.07.001

[ref41] Herrera-Arcos G. Tamez-Duque J. Acosta-De-Anda E. Y. Kwan-Loo K. De-Alba M. Tamez-Duque U. . (2017). Modulation of neural activity during guided viewing of visual art. Front. Hum. Neurosci. 11:581. doi: 10.3389/fnhum.2017.00581, PMID: 29249949 PMC5714858

[ref42] Holochwost S. J. Robb S. L. Henley A. K. Stegenga K. Perkins S. M. Russ K. A. . (2020). Active music engagement and cortisol as an acute stress biomarker in young hematopoietic stem cell transplant patients and caregivers: results of a single case design pilot study. Front. Psychol. 11:587871. doi: 10.3389/fpsyg.2020.587871, PMID: 33224077 PMC7667234

[ref43] Homann K. B. (2010). Embodied concepts of neurobiology in dance/movement therapy practice. Am. J. Dance Ther. 32, 80–99. doi: 10.1007/s10465-010-9099-6, PMID: 40183003

[ref44] Homann K. B. (2017). Engaging the dynamic equilibrium of the mind through dance/movement therapy. Am. J. Dance Ther. 39, 39–42. doi: 10.1007/s10465-017-9251-7, PMID: 40183003

[ref45] Homann K. B. (2020). Dynamic equilibrium: engaging and supporting neurophysiological intelligence through dance/movement therapy. Am. J. Dance Ther. 42, 296–310. doi: 10.1007/s10465-020-09337-4, PMID: 40183003

[ref46] Homer E. S. (2015). Piece work: fabric collage as a neurodevelopmental approach to trauma treatment. Art Ther. 32, 20–26. doi: 10.1080/07421656.2015.992824

[ref47] Hsieh S.-W. Hsiao S.-F. Liaw L.-J. Huang L.-C. Yang Y.-H. (2019). Effects of multiple training modalities in the elderly with subjective memory complaints: a pilot study. Medicine 98:e16506. doi: 10.1097/MD.0000000000016506, PMID: 31335722 PMC6709103

[ref48] Iosa M. Aydin M. Candelise C. Coda N. Morone G. Antonucci G. . (2021). The Michelangelo effect: art improves the performance in a virtual reality task developed for upper limb neurorehabilitation. Front. Psychol. 11:611956. doi: 10.3389/fpsyg.2020.611956, PMID: 33488478 PMC7817887

[ref49] Josef L. Goldstein P. Mayseless N. Ayalon L. Shamay-Tsoory S. G. (2019). The oxytocinergic system mediates synchronized interpersonal movement during dance. Sci. Rep. 9:1894. doi: 10.1038/s41598-018-37141-1, PMID: 30760751 PMC6374432

[ref50] Kaimal G. Ayaz H. Herres J. Dieterich-Hartwell R. Makwana B. Kaiser D. H. . (2017). Functional near-infrared spectroscopy assessment of reward perception based on visual self-expression: coloring, doodling, and free drawing. Arts Psychother. 55, 85–92. doi: 10.1016/j.aip.2017.05.004, PMID: 40182660

[ref51] Kaimal G. Carroll-Haskins K. Mensinger J. L. Dieterich-Hartwell R. Biondo J. Levin W. P. (2020). Outcomes of therapeutic artmaking in patients undergoing radiation oncology treatment: a mixed-methods pilot study. Integr. Cancer Ther. 19, 1–14. doi: 10.1177/1534735420912835, PMID: 32316856 PMC7177989

[ref52] Kaimal G. Carroll-Haskins K. Topoglu Y. Ramakrishnan A. Arslanbek A. Ayaz H. (2022). Exploratory fNIRS assessment of differences in activation in virtual reality visual self-expression including with a fragrance stimulus. Art Ther. 39, 128–137. doi: 10.1080/07421656.2021.1957341

[ref53] Kang S.-J. Kim H.-S. Baek K.-H. (2021). Effects of nature-based group art therapy programs on stress, self-esteem and changes in electroencephalogram (EEG) in non-disabled siblings of children with disabilities. Int. J. Environ. Res. Public Health 18:5912. doi: 10.3390/ijerph18115912, PMID: 34072927 PMC8199280

[ref54] Kang K. Orlandi S. Lorenzen N. Chau T. Thaut M. H. (2022). Does music induce interbrain synchronization between a non-speaking youth with cerebral palsy (CP), a parent, and a neurologic music therapist? A brief report. Dev. Neurorehabil. 25, 426–432. doi: 10.1080/17518423.2022.2051628, PMID: 35341463

[ref55] Kang K. Thaut M. H. (2019). Musical neglect training for chronic persistent unilateral visual neglect post-stroke. Front. Neurol. 10:474. doi: 10.3389/fneur.2019.00474, PMID: 31139135 PMC6517600

[ref56] Kellman J. (1996). Making sense of seeing: autism and David Marr. Visual Arts Res. 22, 76–89.

[ref57] King J. L. Strang C. (2024). Art therapy and the neuroscience of trauma: Theoretical and practical perspectives. 2nd Edn. London: Routledge.

[ref58] Kleinmintz O. M. Goldstein P. Mayseless N. Abecasis D. Shamay-Tsoory S. G. (2014). Expertise in musical improvisation and creativity: the mediation of idea evaluation. PLoS One 9:e101568. doi: 10.1371/journal.pone.0101568, PMID: 25010334 PMC4092035

[ref59] Klorer P. G. (2005). Expressive therapy with severely maltreated children: neuroscience contributions. Art Ther. 22, 213–220. doi: 10.1080/07421656.2005.10129523, PMID: 40101104

[ref60] Kruk K. A. Aravich P. F. Deaver S. P. deBeus R. (2014). Comparison of brain activity during drawing and clay sculpting: a preliminary qEEG study. Art Ther. 31, 52–60. doi: 10.1080/07421656.2014.903826, PMID: 40101104

[ref61] Lobban J. (2014). The invisible wound: veterans’ art therapy. Int. J. Art Ther. 19, 3–18. doi: 10.1080/17454832.2012.725547

[ref62] Malik S. (2022). Using neuroscience to explore creative media in art therapy: a systematic narrative review. Int. J. Art Ther. 27, 48–60. doi: 10.1080/17454832.2021.1998165

[ref9002] Mandić-Gajić G. (2018). The cognitive impairment illustrated in drawings used in gaining insight and motivation in alcoholism treatment. Vojnosanitetski Pregled, 75, 224–227. doi: 10.2298/VSP160210281M

[ref63] McNamee C. M. (2004). Using both sides of the brain: experiences that integrate art and talk therapy through scribble drawings. Art Ther. 21, 136–142. doi: 10.1080/07421656.2004.10129495, PMID: 40101104

[ref64] McNamee C. M. (2005). Bilateral art: integrating art therapy, family therapy, and neuroscience. Contemp. Fam. Ther. 27, 545–557. doi: 10.1007/s10591-005-8241-y

[ref65] Munjuluri S. Bolin P. K. Amy Lin Y. T. Garcia N. L. Gauna L. Nguyen T. . (2020). A pilot study on playback theatre as a therapeutic aid after natural disasters: brain connectivity mechanisms of effects on anxiety. Chronic Stress 4:247054702096656. doi: 10.1177/2470547020966561, PMID: 33210057 PMC7643224

[ref66] Munn Z. Peters M. D. J. Stern C. Tufanaru C. McArthur A. Aromataris E. (2018). Systematic review or scoping review? Guidance for authors when choosing between a systematic or scoping review approach. BMC Med. Res. Methodol. 18:143. doi: 10.1186/s12874-018-0611-x, PMID: 30453902 PMC6245623

[ref67] Netzer D. Brady M. (2009). Parenting as a creative collaboration: a transpersonal approach. J. Creat. Ment. Health 4, 139–151. doi: 10.1080/15401380902945129

[ref68] Oliva A. Iosa M. Antonucci G. De Bartolo D. (2023). Are neuroaesthetic principles applied in art therapy protocols for neurorehabilitation? A systematic mini-review. Front. Psychol. 14:1158304. doi: 10.3389/fpsyg.2023.1158304, PMID: 37377696 PMC10291050

[ref69] Pąchalska M. (2022). Neuropsychology of creativity: a microgenetic approach. Acta Neuropsychol. 20, 87–114. doi: 10.5604/01.3001.0015.8161

[ref70] Pąchalska M. Buliński L. Kaczmarek B. Bach B. Jastrzębowska G. Bazan M. (2013). Fine art and the quality of life of a prominent artist with frontotemporal dementia. Acta Neuropsychol. 11, 451–471. doi: 10.5604/17307503.1090492

[ref9003] Pąchalska M. Góral-Półrola J. (2020). The neuropsychology of metaphors in patients awakened from post-traumatic coma. Acta Neuropsychol. 18, 437–464. doi: 10.5604/01.3001.0014.4987

[ref71] Pąchalska M. Góral-Półrola J. Chojnowska-Ćwiąkała I. (2021). Effect of individually-tailored TDCS and symbolic art therapy for chronic associative prosopagnosia after infection by SARS-COV-2, NEUROCOVID-19 and ischemic stroke. Acta Neuropsychol. 19, 329–345. doi: 10.5604/01.3001.0015.0606, PMID: 40184205

[ref72] Perryman K. Blisard P. Moss R. (2019). Using creative arts in trauma therapy: the neuroscience of healing. J. Ment. Health Couns. 41, 80–94. doi: 10.17744/mehc.41.1.07

[ref73] Peters M. D. J. Marnie C. Tricco A. C. Pollock D. Munn Z. Alexander L. . (2021). Updated methodological guidance for the conduct of scoping reviews. JBI Evid. Implem. 19, 3–10. doi: 10.1097/XEB.0000000000000277, PMID: 33570328

[ref74] Pink S. Mackley K. L. (2014). Re-enactment methodologies for everyday life research: art therapy insights for video ethnography. Vis. Stud. 29, 146–154. doi: 10.1080/1472586X.2014.887266

[ref75] Pongan E. Tillmann B. Leveque Y. Trombert B. Getenet J. C. Auguste N. . (2017). Can musical or painting interventions improve chronic pain, mood, quality of life, and cognition in patients with mild Alzheimer’s disease? Evidence from a randomized controlled trial. J. Alzheimers Dis. 60, 663–677. doi: 10.3233/JAD-170410 28922159

[ref76] Popa L.-C. Manea M. C. Velcea D. Șalapa I. Manea M. Ciobanu A. M. (2021). Impact of Alzheimer’s dementia on caregivers and quality improvement through art and music therapy. Healthcare 9:698. doi: 10.3390/healthcare9060698, PMID: 34207703 PMC8226886

[ref77] Riley S. (2004). The creative mind. Art Ther. J. Am. Art Assoc. 21, 184–190. doi: 10.1080/07421656.2004.10129694

[ref78] Saltzman M. R. Matic M. Marsden E. (2013). Adierian art therapy with sexual abuse and assault survivors. J. Individ. Psychol. 69:223.

[ref79] Schindler M. Maihöfner C. Bolwerk A. Lang F. R. (2015). Does participation in art classes influence performance on two different cognitive tasks? Aging Ment. Health 21, 439–444. doi: 10.1080/13607863.2015.1114587, PMID: 26600170

[ref80] Spring D. (2004). Thirty-year study links neuroscience, specific trauma, PTSD, image conversion, and language translation. Art Ther. 21, 200–209. doi: 10.1080/07421656.2004.10129690

[ref81] Stewart E. G. (2004). Art therapy and neuroscience blend: working with patients who have dementia. Art Ther. 21, 148–155. doi: 10.1080/07421656.2004.10129499

[ref82] Strang C. E. (2024). Art therapy and neuroscience: evidence, limits, and myths. Front. Psychol. 15:1484481. doi: 10.3389/fpsyg.2024.1484481, PMID: 39417019 PMC11480049

[ref83] Tramontano M. De Angelis S. Mastrogiacomo S. Princi A. A. Ciancarelli I. Frizziero A. . (2021). Music-based techniques and related devices in neurorehabilitation: a scoping review. Expert Rev. Med. Devices 18, 733–749. doi: 10.1080/17434440.2021.1947793, PMID: 34162284

[ref84] Tricco A. C. Lillie E. Zarin W. O’Brien K. K. Colquhoun H. Levac D. . (2018). PRISMA extension for scoping reviews (PRISMA-ScR): checklist and explanation. Ann. Intern. Med. 169, 467–473. doi: 10.7326/M18-0850 30178033

[ref85] Vaisvaser S. (2019). Moving along and beyond the spectrum: creative group therapy for children with autism. Front. Psychol. 10:417. doi: 10.3389/fpsyg.2019.00417, PMID: 30914987 PMC6423063

[ref86] Vaisvaser S. (2024). Meeting the multidimensional self: fostering selfhood at the interface of creative arts therapies and neuroscience. Front. Psychol. 15:1417035. doi: 10.3389/fpsyg.2024.1417035, PMID: 39386142 PMC11461312

[ref87] Verna V. De Bartolo D. Iosa M. Fadda L. Pinto G. Caltagirone C. . (2020). Te.M.P.O., an app for using temporal musical mismatch in post-stroke neurorehabilitation: a preliminary randomized controlled study. NeuroRehabilitation 47, 201–208. doi: 10.3233/NRE-203126, PMID: 32741789

[ref88] Walker M. S. Kaimal G. Koffman R. DeGraba T. J. (2016). Art therapy for PTSD and TBI: a senior active duty military service member’s therapeutic journey. Arts Psychother. 49, 10–18. doi: 10.1016/j.aip.2016.05.015

[ref89] Walker M. S. Stamper A. M. Nathan D. E. Riedy G. (2018). Art therapy and underlying fMRI brain patterns in military TBI: a case series. Int. J. Art Ther. 23, 180–187. doi: 10.1080/17454832.2018.1473453, PMID: 40101104

[ref90] Warson E. A. Warson J. S. (2023). Bilateral movement and artmaking: Hemispheric integration across the midline (Mouvement bilatéral et création artistique: Intégration hémisphérique sur la ligne médiane). Can. J. Art Ther. 36, 105–113. doi: 10.1080/26907240.2023.2218727

